# Sustainable Electrosynthesis of Cyclohexanone Oxime through Nitrate Reduction on a Zn–Cu Alloy Catalyst

**DOI:** 10.1021/acscatal.3c05388

**Published:** 2024-02-15

**Authors:** Jonathan Sharp, Anna Ciotti, Hayley Andrews, Shaktiswaran R. Udayasurian, Max García-Melchor, Tengfei Li

**Affiliations:** † School of Chemistry and Environment, Manchester Metropolitan University, Chester Street, Manchester M1 5GD, United Kingdom; ‡ School of Chemistry, CRANN and AMBER Research Centres, Trinity College Dublin, College Green, Dublin 2, Ireland

**Keywords:** nitrate electroreduction, cyclohexanone oxime, C−N bond formation, electrosynthesis, Zn−Cu alloy catalyst, DFT calculations, reaction mechanisms

## Abstract

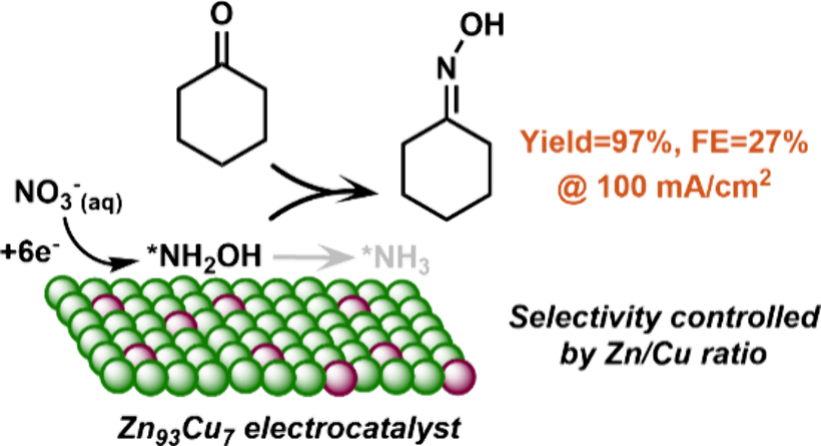

Cyclohexanone oxime is an important precursor for Nylon-6 and is typically synthesized via the nucleophilic addition–elimination of hydroxylamine with cyclohexanone. Current technologies for hydroxylamine production are, however, not environment-friendly due to the requirement of harsh reaction conditions. Here, we report an electrochemical method for the one-pot synthesis of cyclohexanone oxime under ambient conditions with aqueous nitrate as the nitrogen source. A series of Zn–Cu alloy catalysts are developed to drive the electrochemical reduction of nitrate, where the hydroxylamine intermediate formed in the electroreduction process can undergo a chemical reaction with the cyclohexanone present in the electrolyte to produce the corresponding oxime. The best performance is achieved on a Zn_93_Cu_7_ electrocatalyst with a 97% yield and a 27% Faradaic efficiency for cyclohexanone oxime at 100 mA/cm^2^. By analyzing the catalytic activities/selectivities of the different Zn–Cu alloys and conducting in-depth mechanistic studies via *in situ* Raman spectroscopy and theoretical calculations, we demonstrate that the adsorption of nitrogen species plays a central role in catalytic performance. Overall, this work provides an attractive strategy to build the C–N bond in oxime and drive organic synthesis through electrochemical nitrate reduction, while highlighting the importance of controlling surface adsorption for product selectivity in electrosynthesis.

## Introduction

**Figure 1:**
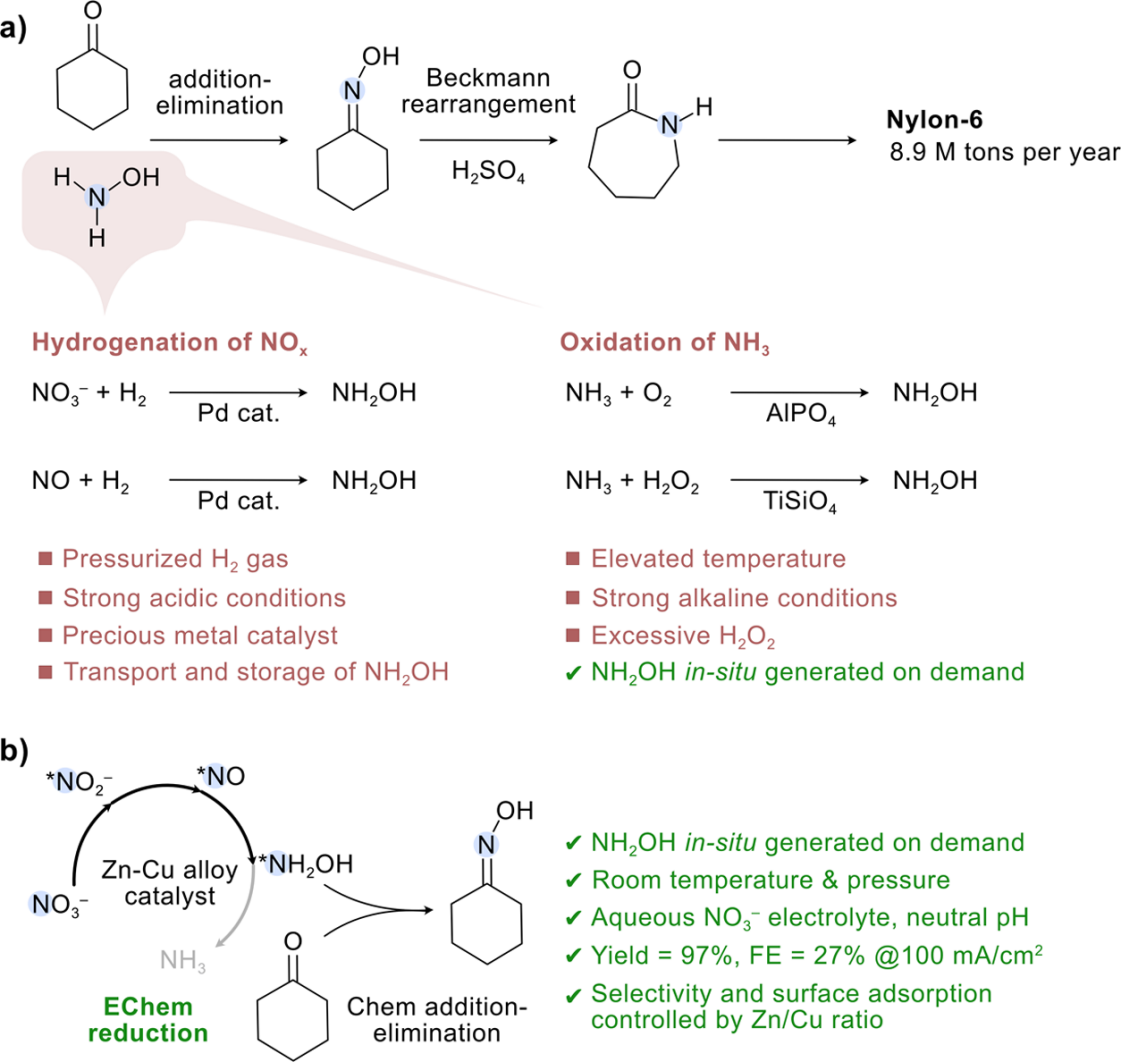
Developments in the synthesis of cyclohexanone oxime via thermal and electrochemical methods. (a) Current technologies to synthesize cyclohexanone oxime from cyclohexanone and hydroxylamine, wherein hydroxylamine production is limited by the harsh reaction conditions. (b) Electrosynthesis of cyclohexanone oxime via nitrate reduction under ambient conditions developed in this work. The surface-adsorbed *NH_2_OH intermediate generated through the electrochemical reduction of nitrate on a Zn–Cu alloy catalyst can undergo a chemical addition–elimination reaction with the cyclohexanone present in the electrolyte to produce oxime.

Cyclohexanone oxime is an important feedstock in the nitrogen industry as it can undergo Beckmann rearrangement to make caprolactam, the monomeric unit of Nylon-6. With the annual global market of Nylon-6 anticipated to be as high as 8.9 million tons by 2024,^1^ the production capacity of cyclohexanone oxime should also be expanded accordingly. To date, the majority of cyclohexanone oxime is produced by the nucleophilic addition–elimination reaction between cyclohexanone and hydroxylamine (NH_2_OH),^2^ which is typically generated via hydrogenation of NO_*x*_ on palladium catalysts^3^ or oxidation of ammonia (NH_3_) by O_2_/H_2_O_2_ (Figure 1a).^4−6^ However, these chemical processes generally require harsh reaction conditions (e.g., high temperatures and/or pressures, strong acidic/alkaline solutions) and therefore are challenged by a series of environmental and safety concerns. More specifically, the hydrogenation route is limited by the requirements of pressurized H_2_ gas, strong acidic conditions and precious metal catalysts. Moreover, transportation and storage of concentrated NH_2_OH also faces challenges, including the risk of explosion.^7^ On the other hand, NH_3_ oxidation utilizes NH_2_OH generated *in situ* as needed.^4^ Yet, this synthetic route still demands elevated temperatures, high pH, and an excess of H_2_O_2_ as the oxidant, which also has transportation and storage issues.^8,9^ Hence, there is a strong desire for an alternative strategy to produce cyclohexanone oxime under mild conditions.

Electrosynthesis stands out as a promising synthetic strategy to enable the sustainable production of chemical feedstocks and added-value compounds by driving reactions under ambient conditions, using electrons generated from renewable electricity instead of redox reagents.^10−14^ Particularly, electrochemical nitrate reduction (NO_3_R) has been demonstrated as a sustainable synthetic approach to upgrade nitrate waste into NH_3_, a very important chemical building block, thus contributing to abating the global nitrogen cycle imbalance.^15−23^ Furthermore, a series of surface-adsorbed reaction intermediates have been identified in the NO_3_R to ammonia, such as *NO, *NH_2_OH, and *NH_2_, which exhibit high nucleophilic reactivities and therefore can be coupled with chemical reactions to build C–N bonds.^24,25^ In particular, the electrochemical coreduction of CO_2_ and NO_3_^–^ has been reported as an attractive route to produce organonitrogen compounds, including urea, methylamine, and glycine.^26−30^ Recently, the Zhang and Tan groups have also shown that the *NH_2_ intermediate generated during NO_3_R can interact with CO_2_-derived compounds (i.e., formic acid or CO) to produce formamide.^31,32^ Despite these distinctive methods, current studies on C–N bond formation via NO_3_R mainly focus on coupling NO_3_R intermediates with C_1_–C_2_ molecules (e.g., CO_2_, CO, formic acid, oxalic acid). There are a few recent reports on electrochemical NO_3_R systems, which can build C–N bonds in larger organic molecules (i.e., C_3+_ compounds), such as amino acid^33,34^ and oxime.^35−38^ However, NO_3_R can generate a variety of N-containing byproducts (e.g., N_2_, NH_3_, N_2_O, NO_2_^–^), and therefore the understanding of the intrincate NO_3_R reaction pathways and the controlling of N selectivity toward the desired organonitrogen products remain elusive. Furthermore, the adsorption of N species on the electrocatalyst surface, which plays a central role in tuning the product selectivity, is still largely unexplored for building C–N bonds in C_3+_ molecules.

Previous reports revealed that the surface-adsorbed *NH_2_OH is a key intermediate in the NO_3_R to NH_3_.^17,18,28,39,40^ Recently, the groups of Zhang, Zou, and Li have also independently reported that the *NH_2_OH intermediate produced during the electrochemical reduction of NO_3_^–^/NO_2_^–^/NO can undergo a C–N coupling reaction to form oxime.^36−38^ Herein, we report a two-step electrochemical-chemical (EChem–Chem) process for the one-pot synthesis of cyclohexanone oxime with aqueous nitrate as the N source (Figure 1b) and provide an in-depth investigation of how the product selectivity is controlled by the surface adsorption of N species. A series of Zn–Cu alloys are prepared to drive the electrochemical NO_3_R step and generate *NH_2_OH, which can then undergo a chemical reaction with cyclohexanone in solution to produce oxime. Through a combination of experimental and computational studies, we demonstrate that the binding of the reaction intermediates on the electrocatalyst surface plays a central role in the EChem–Chem process. Particularly, we show that weak surface adsorption (e.g., on a pure Zn catalyst) can lead to high potentials for the EChem step, whereas strong adsorption (e.g., on a pure Cu catalyst) can promote this process at lower potentials but results in the complete reduction of *NH_2_OH to NH_3_ instead of forming oxime. Notably, we find that a Zn–Cu alloy catalyst with an optimized composition of Zn_93_Cu_7_ can efficiently convert aqueous NO_3_^–^ (pH 7.0) and cyclohexanone into cyclohexanone oxime with a yield of 97 ± 2% and a Faradaic efficiency (FE) of 27 ± 2% at 100 mA/cm^2^. The NH_2_OH-mediated reaction pathway and the relationship between oxime-making activity and surface adsorption are supported by *in situ* Raman spectroscopy and theoretical calculations. Altogether, this work represents a novel strategy for the one-pot electrosynthesis of cyclohexanone oxime from aqueous NO_3_^–^ under ambient conditions, which can upgrade NO_3_^–^ and enable sustainable organic synthesis. This study also highlights the potential of producing value-added organonitrogen compounds via electrochemical NO_3_R as well as the importance of controlling the binding of surface species for product selectivity in electrocatalysis.

## Results and Discussion

### Preparation and Structural Characterization of the Zn_93_Cu_7_ Electrocatalyst

**Figure 2:**
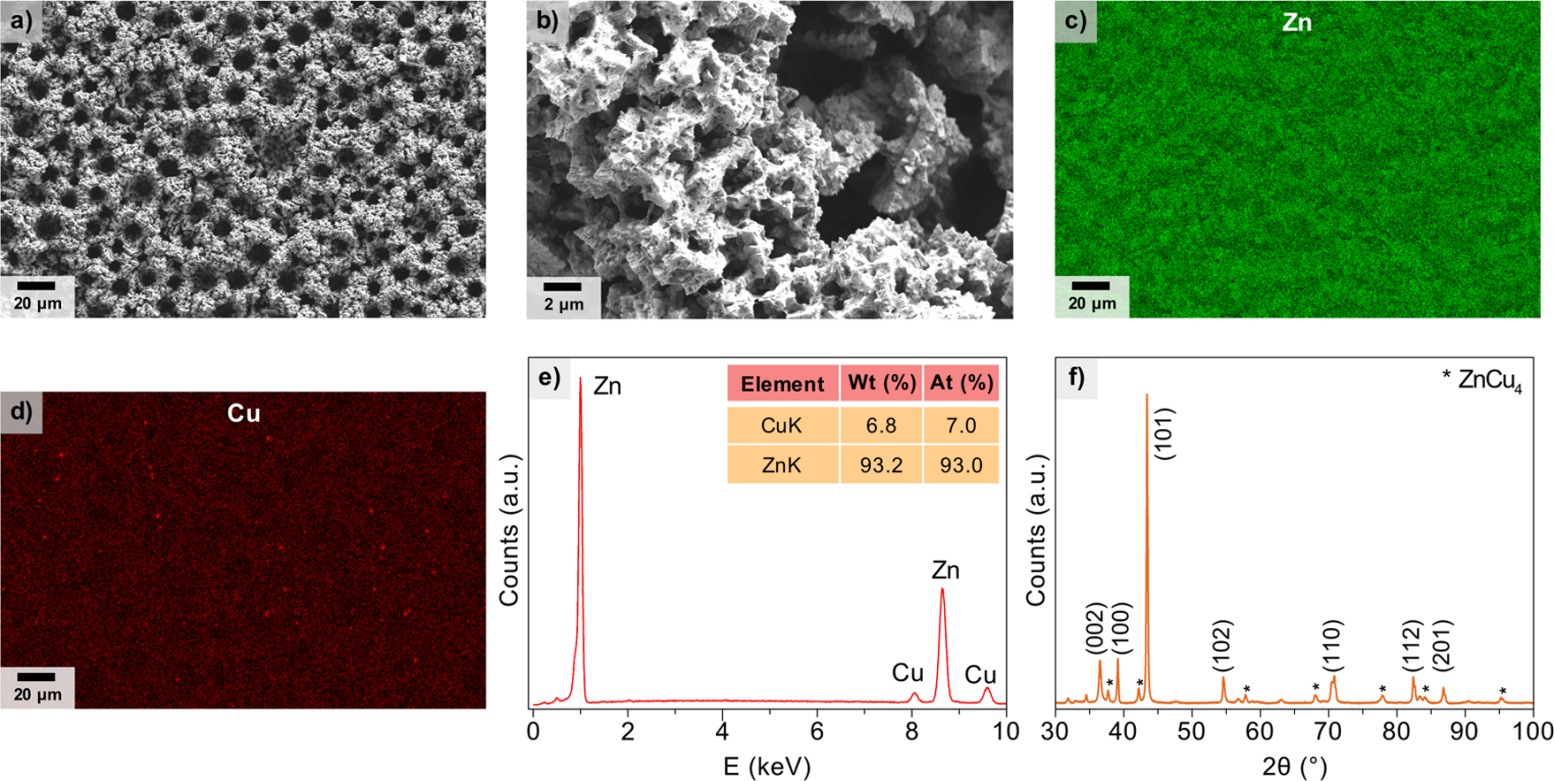
Structural characterization of the Zn_93_Cu_7_ electrocatalyst. (a, b) SEM images at different magnifications. (c) EDX mapping for Zn. (d) EDX mapping for Cu. (e) EDX quantitative elemental analysis. (f) XRD pattern. The planes in brackets are attributed to Zn (JCPDS 04–0831) and the planes with stars correspond to ZnCu_4_ phase (JCPDS65–6066).

A Zn_93_Cu_7_ electrocatalyst was prepared via electrodeposition. Previous reports have demonstrated that electrodeposition of metal precursors in acid solution can generate metal films (e.g., Cu, Ag, Pd, Zn, and Sn) with highly porous surface caused by the formation of H_2_ bubbles during the process, known as the dynamic hydrogen bubble template (DHBT) approach.^41−43^ Here, we utilize the DHBT method to prepare a Zn–Cu alloy catalyst, where Zn^2+^ and Cu^2+^ precursors (Zn^2+^/Cu^2+^ ratio = 60) in aqueous acidic solution were electrochemically reduced and deposited onto a Cu foil substrate to form an alloy film (see Supporting Information). Scanning electron microscopy (SEM) of the Zn–Cu alloy (Figure 2a,b) showed a uniform microporous morphology that is typically observed in metal films prepared by DHBT.

Energy-dispersive X-ray (EDX) mapping showed a homogeneous elemental distribution of Zn and Cu in the sample (Figure 2c,d), indicating the successful formation of an alloy. Furthermore, EDX quantitative analysis revealed a composition of 93 atom % and 7 atom % for Zn and Cu, respectively (Figure 2e). Inductively coupled plasma optical emission spectrometry (ICP-OES) showed a similar elemental composition for the bulk catalyst of 91% Zn and 9% Cu (Table S1). As the electrocatalytic activity and reaction pathway are mainly determined by the surface composition, instead of bulk composition, hereafter we refer to this alloy as Zn_93_Cu_7_. The successful formation of the alloy was also confirmed by X-ray diffraction (XRD), which mainly exhibited crystal planes of pure Zn and minor peaks of a ZnCu_4_ phase (Figure 2f), with no evidence of pure Cu. Hence, the XRD results indicate that Cu was homogeneously distributed within the Zn matrix, in agreement with EDX mapping.

### Electrosynthesis of Cyclohexanone Oxime by Zn_93_Cu_7_

The Zn_93_Cu_7_ alloy (surface area = 1 cm^2^) was used as working electrode to drive the electrochemical NO_3_R to produce cyclohexanone oxime in 16 mL of 0.5 M aqueous potassium phosphate (KPi) buffer solution (pH 7.0) containing 100 mM KNO_3_ and 25 mM cyclohexanone. Pt and Ag/AgCl electrodes were used as counter and reference electrodes, respectively, and electrochemical tests were carried out in a gastight H-type cell at constant current (chronopotentiometry). More details of the electrochemical cell setup can be found in Experimental methods and Figure S1. Electrolysis time was adjusted to keep a constant value of charges injected into the electrolyte. The potential required to achieve a certain current density was obtained from the steady-state potential of the electrolysis. A chronopotentiometry graph for electrolysis over 2.5 h at 100 mA/cm^2^ can be found in Figure S2 as an example of obtaining the steady-state potential.

**Figure 3:**
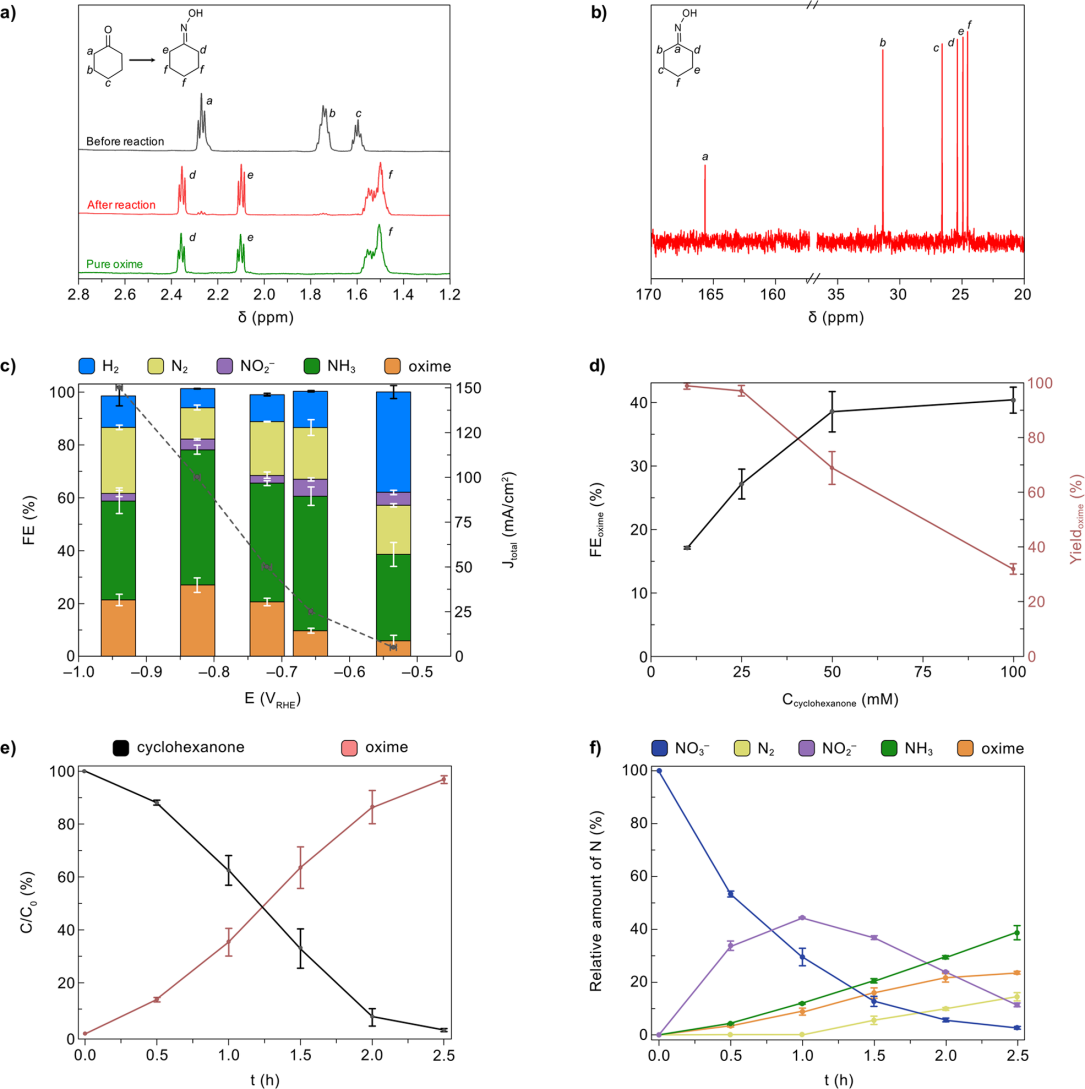
Electrosynthesis of cyclohexanone oxime from NO_3_^–^and cyclohexanone driven by a Zn_93_Cu_7_electrocatalyst. (a) ^1^H NMR spectra for the reaction mixture before and after 2.5 h electrolysis, and pure cyclohexanone oxime as control. (b) ^1^C NMR spectra for the reaction mixture after reaction. (c) FE and total current density (*J*_total_) at different potentials. Potential values were obtained from the steady-state potentials for constant current electrolysis. (d) FE and yield of cyclohexanone oxime measured at 100 mA/cm^2^ with different concentrations of cyclohexanone added into 100 mM KNO_3_ electrolyte. (e) Conversion of cyclohexanone into cyclohexanone oxime over 2.5 h of electrolysis at 100 mA/cm^2^. (f) Relative amount of nitrogen products over 2.5 h of electrolysis at 100 mA/cm^2^. Reaction conditions: Zn_93_Cu_7_ cathode (surface area = 1 cm^2^) immersed in 16 mL of aqueous buffer solution (0.5 M KPi, pH 7.0) containing 100 mM KNO_3_ and 25 mM cyclohexanone. Error bars correspond to the standard deviation of triplicate experiments.

The liquid reaction mixture was analyzed by nuclear magnetic resonance (NMR) to identify the organic compounds present. The recorded ^1^H NMR spectra before and after electrolysis, shown in Figure 3a, indicate that the cyclohexanone peaks almost disappeared after the reaction, while new peaks aligned with pure cyclohexanone oxime emerged, confirming the successful conversion of cyclohexanone into cyclohexanone oxime. The ^13^C NMR spectrum obtained for the reaction mixture after electrolysis also supported the formation of cyclohexanone oxime (Figure 3b).

The production of cyclohexanone oxime was further confirmed by mass spectroscopy (MS, Figure S3). The reaction mixture was also analyzed by high-performance liquid chromatography (HPLC) to quantify cyclohexanone and cyclohexanone oxime (Figure S4) as well as ion chromatography (IC) to detect and quantify different nitrogen species in solution, namely, NO_3_^–^, NO_2_^–^, and NH_3_ (Figure S5). In addition, the gas sample in the headspace of the H-cell was analyzed by gas chromatography (GC). Details of products characterization can be found in the Supporting Information.

The FE and steady-state potential for the electrochemical NO_3_R were measured at constant current densities of 5, 25, 50, 100, and 150 mA/cm^2^, the results of which are summarized in Figure 3c. At 5 mA/cm^2^, a small amount of cyclohexanone oxime was detected (FE = 6 ± 2%), while the major products were NH_3_ and H_2_. The FE for cyclohexanone oxime increased to 27 ± 2% at 100 mA/cm^2^ and −0.82 V vs reversible hydrogen electrode (V_RHE_) and then decreased to 21 ± 2% when the current density further increased to 150 mA/cm^2^. At the different current densities investigated in this work, NH_3_ was generated as the major product with FEs between 30 and 50%; other detected products included NO_2_^–^, N_2_, and H_2_.

The influence of different concentrations of cyclohexanone (i.e., 10, 25, 50, 100 mM; NO_3_^–^ concentration was kept constant at 100 mM) added to the electrolyte was investigated through 2.5 h electrolysis experiments at 100 mA/cm^2^ (Figure 3d). Low concentrations of cyclohexanone (e.g., 10 mM) were found to produce cyclohexanone oxime with high yields (99 ± 1%), while the FE for cyclohexanone oxime was quite low (17 ± 0.3%). In contrast, high concentrations of cyclohexanone (e.g., 100 mM) led to high FE for cyclohexanone oxime (40 ± 2%), but the yield was lower (32 ± 2%). Therefore, a medium concentration of cyclohexanone (25 mM) was chosen to balance the yield and FE for cyclohexanone oxime.

After optimizing the reaction conditions as 100 mA/cm^2^ and 25 mM cyclohexanone, we next monitored the temporal changes in the concentrations of the reactants and products by analyzing reaction aliquots every 30 min over 2.5 h of electrolysis. As shown in Figure 3e, oxime was produced with 97 ± 2% yield and the ketone reactant was almost fully converted after 2.5 h (conversion ∼98%), which is consistent with the recorded ^1^H NMR spectrum. These results also demonstrated a high carbon selectivity which is close to 100%, while the nitrogen selectivity was investigated by monitoring the relative amounts of all nitrogen compounds over the 2.5 h electrolysis (Figure 3f). The NO_3_^–^ reactant was first converted into NO_2_^–^, which achieved a maximum yield of ca. 45% after 1 h, indicating that NO_2_^–^ is a reaction intermediate, in line with previous reports.^15−20,44^ After 2.5 h of electrolysis, ca. 3% NO_3_^–^ and 15% NO_2_^–^ remained in solution, while cyclohexanone oxime and NH_3_ were the main species produced in ca. 24% and 40% yields, respectively. Under these conditions, N_2_ was generated as a byproduct (∼15%), which is consistent with previous NO_3_R studies driven by Zn-rich electrocatalysts.^45−47^ Importantly, the surface structures of the Zn_93_Cu_7_ electrocatalyst remained stable after three tests of 2.5 h electrolysis (Figures S6 and S7), and the electrocatalyst could be reused to produce cyclohexanone oxime with similar FEs compared to the initial test (Figure S8).

Next, a series of control experiments were performed to confirm the C and N sources and shed light into the reaction pathway (Table 1). As expected, the reaction in the absence of electrolysis or cyclohexanone or NO_3_^–^ did not yield any oxime product (entries 2, 3 and 4). Experiments using bare Cu foil as the cathode (entry 5) were also inactive toward oxime formation, highlighting the catalytic role of the Zn_93_Cu_7_ alloy. To confirm the N source, an isotopic labeling experiment with K^15^NO_3_ was carried out (entry 6), and the formation of ^15^N-labeled cyclohexanone oxime was corroborated by mass spectroscopy (Figure S9). The recorded ^1^H NMR spectrum (Figure S9) also revealed the duplet splitting behavior of the ^15^NH_4_^+^ peak generated from ^15^NO_3_^–^ reduction, while ^14^NH_4_^+^ produced by normal ^14^NO_3_^–^ reduction features a triplet peak. A series of possible NO_3_R intermediates, including NO_2_^–^, NO, and NH_2_OH, was each used as the starting N source (entries 7–9) and resulted in the formation of cyclohexanone oxime, indicating a NO_3_R reaction pathway via those species that is consistent with previous studies.^17,18,28^ Finally, using NH_3_ as the N source (entry 10) failed to produce any cyclohexanone oxime, further confirming that the oxime product was generated from an intermediate of NO_3_R instead of the final product of NO_3_R (NH_3_).

**Table 1:** Control Experiments for Cyclohexanone Oxime Production

**entry**	**cathode**	**C source**	**N source**	**electrolysis**	**cyclohexanone oxime?**
1	Zn_93_Cu_7_	cyclohexanone	KNO_3_	yes	yes
2	Zn_93_Cu_7_	cyclohexanone	KNO_3_	no	no
3	Zn_93_Cu_7_		KNO_3_	yes	no
4	Zn_93_Cu_7_	cyclohexanone		yes	no
5	Cu foil	cyclohexanone	KNO_3_	yes	no
6	Zn_93_Cu_7_	cyclohexanone	K^15^NO_3_	yes	yes, ^15^N-labeled
7	Zn_93_Cu_7_	cyclohexanone	KNO_2_	yes	yes
8	Zn_93_Cu_7_	cyclohexanone	NO	yes	yes
9	Zn_93_Cu_7_	cyclohexanone	NH_2_OH	yes	yes
10	Zn_93_Cu_7_	cyclohexanone	NH_3_	yes	no

### Electrosynthesis of Cyclohexanone Oxime by Different Zn–Cu Alloys

**Figure 4:**
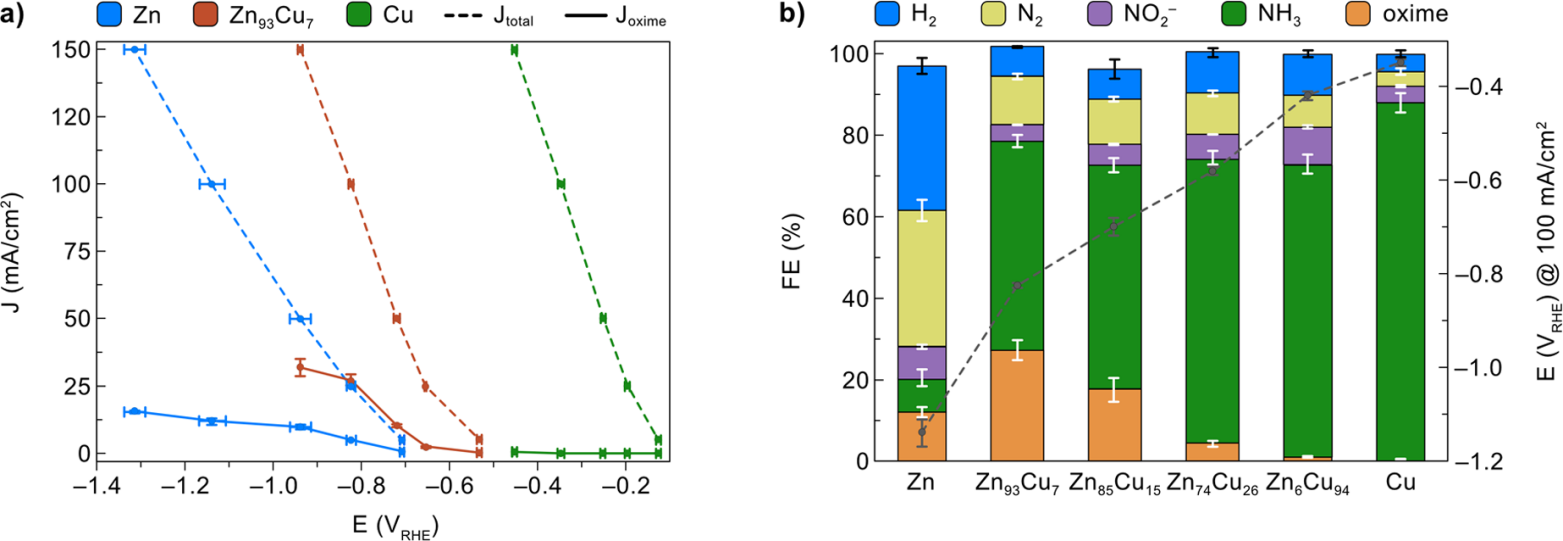
Production of cyclohexanone oxime from NO_3_^–^and cyclohexanone driven by different Zn/Cu electrocatalysts. (a) Total current density (*J*_total_) and partial current density for cyclohexanone oxime (*J*_oxime_) at different potentials for Zn, Zn_93_Cu_7_, and Cu electrocatalysts. (b) FE and potential required to achieve *J*_total_ at 100 mA/cm^2^ with different Zn/Cu electrocatalysts. Reaction conditions: Zn/Cu cathode (surface area = 1 cm^2^) immersed in 16 mL of aqueous buffer solution (0.5 M KPi, pH 7.0) containing 100 mM KNO_3_ and 25 mM cyclohexanone. Potential values were obtained from the steady-state potentials for constant current electrolysis. Error bars correspond to the standard deviation of triplicate experiments.

Pure Zn and Cu electrocatalysts were also prepared through similar DHBT electrodeposition methods to compare their catalytic performance with that of the Zn_93_Cu_7_ alloy. The X-ray photoelectron spectroscopy (XPS) analysis revealed that the Zn/Cu catalysts were mainly in metallic phases while a small portion of metal oxide was formed due to the inevitable surface oxidation of the catalysts in the atmosphere (Figure S10). The SEM images and XRD results for pure Zn and Cu catalysts are also shown in Figures S11 and S12. As displayed in Figure 4a, pure Cu required a significantly lower potential than pure Zn to achieve the same total current density (*J*_total_, top), while the Zn_93_Cu_7_ alloy showed an intermediate behavior, suggesting that the electrochemical NO_3_R is more favorable on Cu. However, pure Cu could barely produce any cyclohexanone oxime, whereas pure Zn was capable of forming cyclohexanone oxime. Notably, Zn_93_Cu_7_ attained an even higher partial current density for oxime (*J*_oxime_, bottom) at a significantly lower potential compared to pure Zn. For example, a *J*_oxime_ of 32 ± 3 mA/cm^2^ was achieved at −0.94 V_RHE_ on Zn_93_Cu_7_, compared to 10 ± 1 mA/cm^2^ on pure Zn.

Although pure Cu itself was deemed inactive toward cyclohexanone oxime production, the introduction of a small percentage (7%) of Cu into Zn resulted in a significantly enhanced performance. Hence, to investigate how Zn/Cu composition influences NO_3_R, a series of Zn–Cu alloy catalysts were prepared by electrodeposition using precursor solutions with different Zn/Cu ratios (see Table S1 for details). The bulk composition was measured by ICP-OES analysis (Table S1). EDX analysis of the surface elemental composition (Figure S13), which is more related to the electrocatalytic activity, determined the surface composition of these samples to be Zn_93_Cu_7_, Zn_85_Cu_15_, Zn_74_Cu_26_, and Zn_6_Cu_94_. Subsequently, the Zn–Cu alloys were tested for NO_3_R at 100 mA/cm^2^, with the measured potentials and FEs summarized in Figure 4b. As the Cu content increased, the NO_3_R could be driven at lower potentials and the FE for NH_3_ was significantly enhanced, consistent with previous reports that Cu is an efficient electrocatalyst for NO_3_^–^ to NH_3_ conversion.^15,17,18^ However, Cu-rich catalysts (i.e., Zn_6_Cu_94_ and pure Cu) resulted in low FEs for cyclohexanone oxime (<1%). The highest FE for the oxime product was achieved on Zn_93_Cu_7_ (27 ± 3%), while pure Zn and Zn_85_Cu_15_ produced oxime with lower FEs (12 ± 1% and 17 ± 3%, respectively). We also note that pure Zn also produced a substantial amount of N_2_ (FE = 33 ± 3%) and H_2_ (FE = 35 ± 2%) compared to the other electrocatalysts.

Based on the above experimental results and previous NO_3_R studies^48^ that revealed a significantly stronger adsorption of reaction intermediates on Cu compared to Zn, we posited four possible (not excluding) reasons for the different behavior observed with the Zn–Cu alloy electrocatalysts: (1) Cu has a stronger surface adsorption of NO_3_^–^ than Zn and therefore facilitates the electrochemical NO_3_R at lower potentials; (2) Cu favors the further electroreduction of *NH_2_OH to NH_3,_ instead of the chemical reaction with cyclohexanone to give oxime; (3) introducing Cu into Zn creates a more porous surface morphology and thus leads to lower NO_3_R potentials; (4) pure Zn is disadvantaged by the competing NO_3_R pathway toward N_2_, rather than NH_2_OH or NH_3_. The difference in surface morphology for Zn, Cu, and Zn_93_Cu_7_ is supported by SEM images (Figure S11) and the measurements of the electrochemical active surface area (ECSA, Figure S14). Hence, we next carried out further experimental and theoretical studies to prove the other three possible reasons.

### Experimental Mechanistic Studies

**Figure 5:**
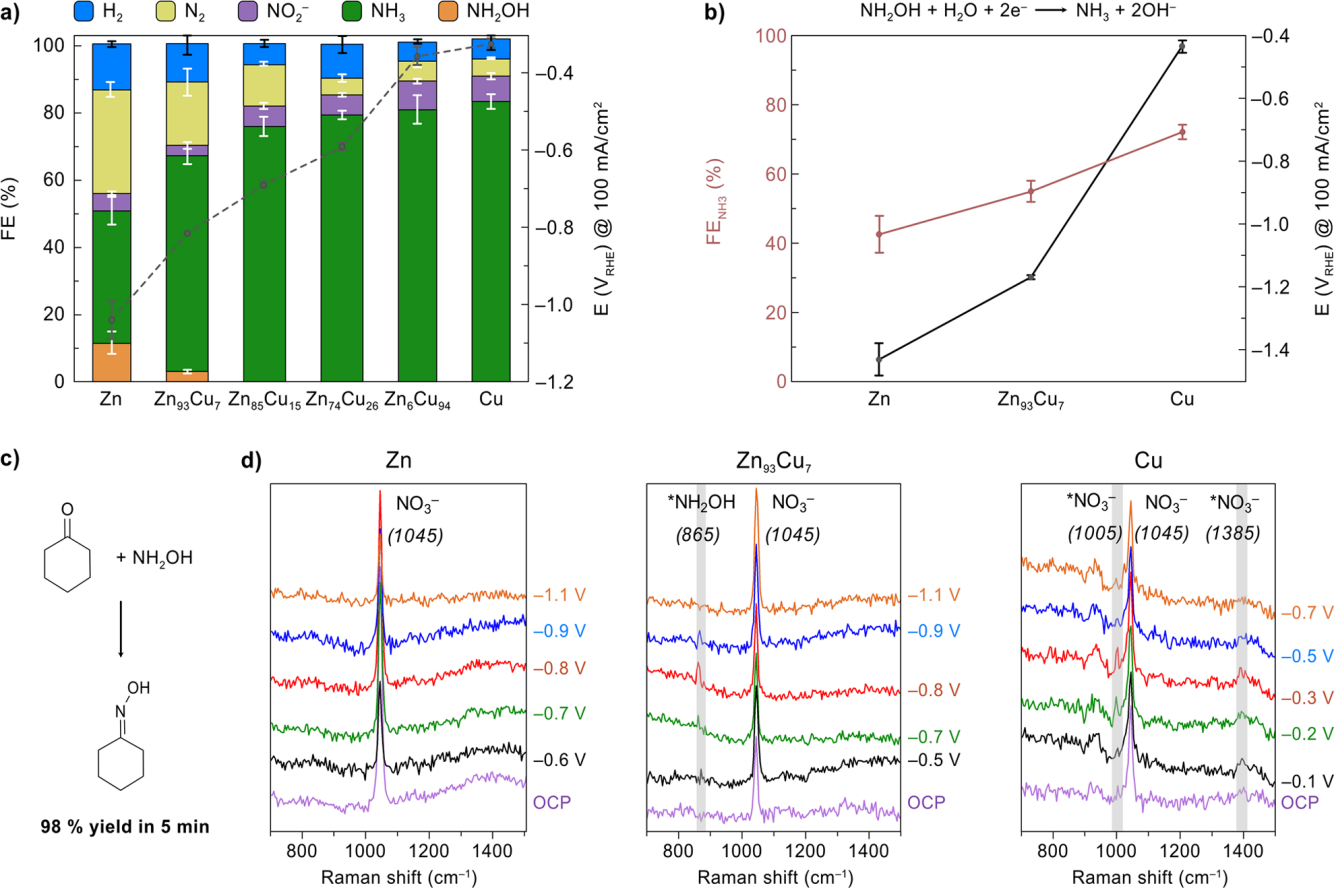
Mechanistic study of the NO_3_R process mediated by surface-adsorbed NH_2_OH. (a) Electroreduction of NO_3_^–^ (without cyclohexanone) at 100 mA/cm^2^ with different Zn–Cu alloy catalysts. (b) Electroreduction of NH_2_OH to NH_3_ at 100 mA/cm^2^ for Zn, Zn_93_Cu_7_ and Cu. Reaction conditions: Zn/Cu cathode (surface area = 1 cm^2^) immersed in 16 mL aqueous buffer solution (0.5 M KPi, pH 7.0) containing 100 mM KNO_3_ (for a) or NH_2_OH (for b). Error bars correspond to the standard deviation of triplicate experiments. (c) Chemical addition–elimination reaction between cyclohexanone and NH_2_OH to yield cyclohexanone oxime. Reaction conditions: 16 mL aqueous solution containing 25 mM cyclohexanone and 25 mM NH_2_OH. (d) *In situ* Raman spectra for electrochemical NO_3_R (without cyclohexanone) recorded at different potentials (vs RHE) on pure Zn, Zn_93_Cu_7_ alloy, and pure Cu electrocatalysts. The peak at 1045 cm^–1^ was consistently observed for the NO_3_^–^ in solution. The asterisk in the labeled species denote an adsorbed state, e.g., *NH_2_OH, *NO_3_^–^.

The detection of the NH_2_OH intermediate proved very challenging in the presence of cyclohexanone because of the extremely fast nucleophilic addition of NH_2_OH to the ketone. Therefore, we set out to perform electrochemical NO_3_R experiments with the different Zn/Cu catalysts in the absence of cyclohexanone to confirm the formation of NH_2_OH. As shown in Figure 5a, similar trends of lower potentials and higher FE toward NH_3_ were observed as the Cu content increased, consistent with hypothesis 1 that Cu facilitates electrochemical NO_3_R. More importantly, pure Zn and Zn_93_Cu_7_ were found to produce NH_2_OH at 100 mA/cm^2^ with FEs of 12 ± 3% and 3 ± 1%, respectively (detection of NH_2_OH in solution is shown in Figure S15). Pure Zn can produce more NH_2_OH than Zn_93_Cu_7_ in the absence of cyclohexanone because the *NH_2_OH produced on Zn_93_Cu_7_ can be further reduced to NH_3_ when cyclohexanone is unavailable (see below discussion that Zn_93_Cu_7_ is a better NH_2_OH electroreduction catalyst than pure Zn). Conversely, in the presence of cyclohexanone, Zn_93_Cu_7_ gave more oxime because the addition of cyclohexanone shifts the equilibrium toward cyclohexanone oxime formation, outcompeting *NH_2_OH reduction. The formation of *NH_2_OH and its subsequent release into the electrolyte solution were also evidenced at other current densities for Zn_93_Cu_7_ (Figure S16). In contrast, the other electrocatalysts with higher Cu contents did not yield any detectable amount of NH_2_OH, indicating that this intermediate can be further reduced to NH_3_ on Cu-rich catalysts, whereas on Zn-rich catalysts (i.e., pure Zn and Zn_93_Cu_7_) the *NH_2_OH intermediate can desorb from the surface and accumulate in the solution to a detectable concentration.

To further understand the interaction of NH_2_OH with the electrocatalyst surface, the electroreduction of NH_2_OH to NH_3_ was investigated on pure Zn, Zn_93_Cu_7_, and pure Cu (Figure 5b). Experiments with pure Cu led to a significantly higher FE for NH_3_ and a lower potential compared to pure Zn and Zn_93_Cu_7_, demonstrating that the electroreduction of NH_2_OH to NH_3_ is much more favorable on the Cu surface compared to Zn. The central role of NH_2_OH in the production of oxime was further confirmed by mixing cyclohexanone and NH_2_OH in solution without electrolysis, which readily produced cyclohexanone oxime with 98% yield after only 5 min (Figure 5c). The fast kinetics and favorable thermodynamics of this noncatalytic addition–elimination reaction ensures effective C–N bond formation. Given that NH_2_OH is the most nucleophilic species among all the proposed NO_3_R intermediates and the widely known nucleophilic addition of NH_2_OH to ketones,^28^ we concluded that NH_2_OH is the NO_3_R intermediate that reacts with cyclohexanone to generate oxime. These results collectively support hypothesis 2 that, on Zn-rich electrocatalysts, the electrochemically generated NH_2_OH tends to undergo a subsequent chemical reaction with cyclohexanone to produce oxime rather than being further reduced to NH_3_.

Further insights were obtained by analyzing the surface-adsorbed species during electrochemical NO_3_R at different potentials by *in situ* Raman spectroscopy (Figure 5d). Experiments with pure Zn showed a single Raman peak at 1045 cm^–1^, which was attributed to the NO_3_^–^ in solution^49,50^ (a control experiment with an aqueous KNO_3_ solution in absence of electrocatalyst also showed the peak at 1045 cm^–1^). On the other hand, for Zn_93_Cu_7_, a small peak at 865 cm^–1^ was observed at −0.5 V_RHE_, which gradually grew as the potential increased to −0.8 V_RHE_ before disappearing at −1.1 V_RHE_. This peak can be attributed to the N–O stretch mode of surface-adsorbed *NH_2_OH intermediate,^49,51^ providing direct experimental evidence for this species on the Zn_93_Cu_7_ surface. This observation also confirms hypothesis 2 that Zn_93_Cu_7_ can generate *NH_2_OH intermediate which plays a central role in the reaction. In contrast, the pure Cu electrocatalyst exhibited two peaks at 1005 and 1385 cm^–1^ corresponding to adsorbed *NO_3_^–^ species,^49,50^ which grew with the potential increasing to −0.3 V_RHE_ and then disappeared at more negative potentials. The absence of the *NH_2_OH peak on pure Cu can be explained by the favored further reduction of *NH_2_OH into NH_3_ on Cu. The absence of a clear Raman peak for the adsorbed *NH_2_OH on pure Zn can be explained by the weak adsorption of *NH_2_OH on Zn, which makes the surface-adsorbed intermediates difficult to be observed by a surface-enhanced technique. Overall, these observations on pure Cu and the absence of adsorbed species on pure Zn collectively support hypothesis 1 that Cu provides a stronger binding for NO_3_^–^ compared to Zn, which is beneficial for electrochemical NO_3_R.

### Computational Mechanistic Studies

To shed further light on the different NO_3_R selectivities toward NH_2_OH, NH_3_, and N_2_ observed with the Cu, Zn, and Zn_93_Cu_7_ electrocatalysts, we performed a mechanistic investigation by means of periodic density functional theory (DFT) calculations (see the Supporting Information for details). To avoid the well-documented issues associated with the modeling of charged species through the introduction of a background charge in the unit cell,^52,53^ and since the reduction from NO_3_^–^ to nitric oxide (NO) is widely understood,^15,37,52−54^ we set the zero of energies in our calculations to be the neutral NO molecule instead of NO_3_^–^ (see the Supporting Information for further details).

To study the NO_3_R reactivity on the different electrocatalysts, we constructed Cu(111), Zn(101), and Zn_93_Cu_7_(101) surface slab models (as confirmed by XRD in Figure 2f and Figure S12) followed by the assessment of their surface coverages under experimental conditions,^55,56^ as depicted in Figure S17. For Cu(111), DFT simulations predict 75% of the *fcc* sites to be covered by H atoms, while all the *bridge* positions on Zn(101) and Zn_93_Cu_7_(101) are occupied by H. Taking these surface terminations as the catalyst resting state, the electroreduction of NO to N_2_, NH_2_OH, and NH_3_ was then explored via a series of proton-coupled electron transfer (PCET) steps, as reported elsewhere.^17,18,28^ Furthermore, at each step, the hydrogenation of both the N and O atoms was considered as well as all the possible adsorption sites for each reaction intermediate (see Tables S2 and S3).

First, the N_2_ evolution reaction was investigated via the mechanism outlined below (* denotes a surface site), which involves the adsorption of NO on the electrode surface, two sequential PCETs at the O atom, and the N–N coupling followed by N_2_ desorption (eqs 1–4).^21,57^
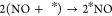
1
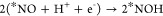
2
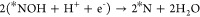
3
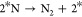
4

**Figure 6:**
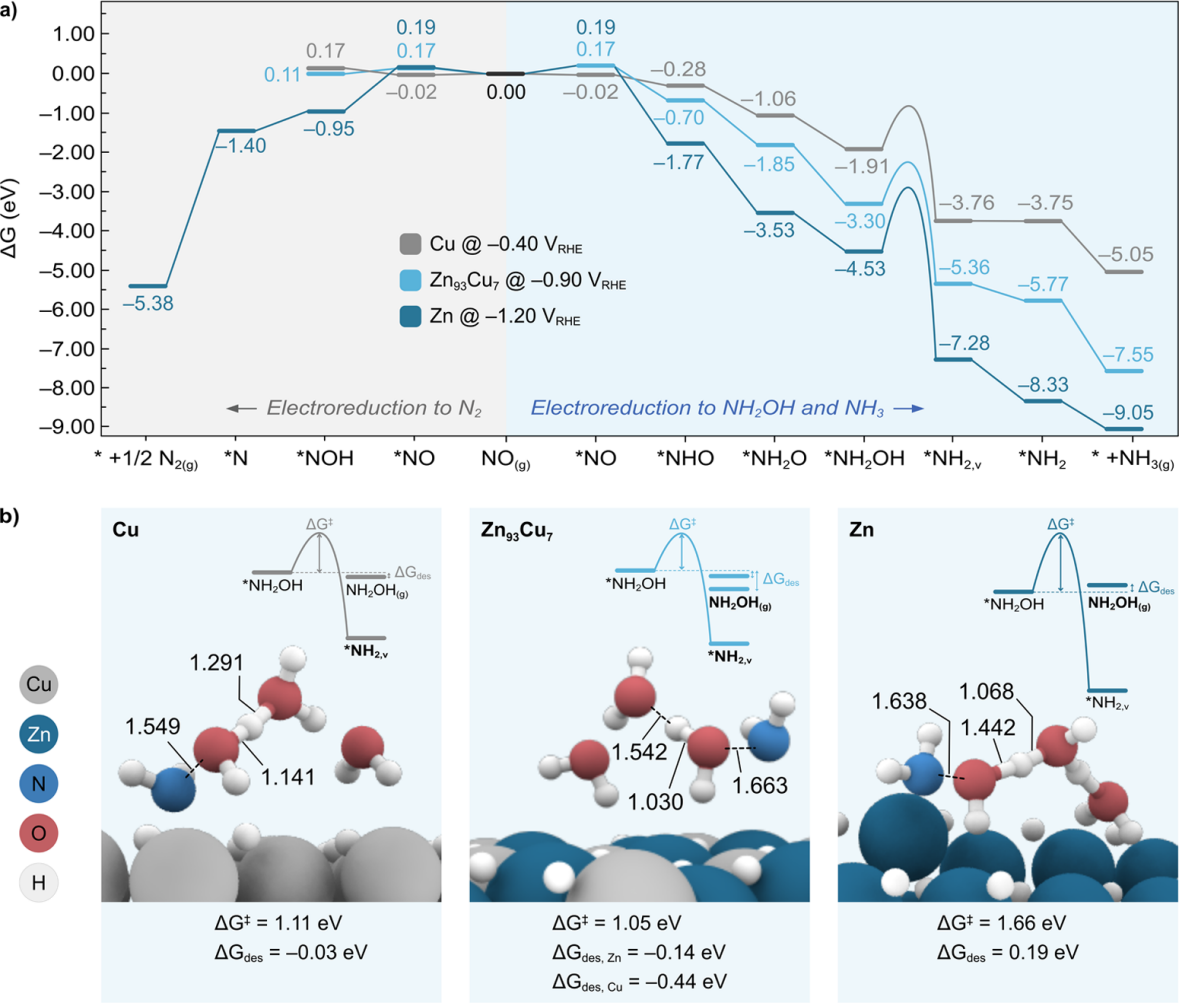
Computational mechanistic studies for the formation of N_2_, NH_2_OH, and NH_3_. (a) Gibbs energy diagram for the electroreduction of NO to N_2_ (left) and NO to NH_2_OH and NH_3_ (right) on the Cu(111), Zn_93_Cu_7_(101) and Zn(101) surfaces at the experimental applied potentials of −0.40, −0.90, and −1.20 V_RHE_, respectively. The label *NH_2,v_ denotes the formation of the *NH_2_ intermediate from *NH_2_OH by sourcing the H atom from the surface coverage, whereas the label *NH_2_ indicates the same intermediate after replenishing the surface H vacancy. (b) Optimized transition state structures and associated kinetic barriers (Δ*G*^‡^) for the electroreduction of *NH_2_OH on pure Cu (left), Zn_93_Cu_7_ (middle), and pure Zn (right). This step was modeled with two explicit water molecules to assist H transfer. Relevant bond distances are given in Å. The Gibbs energies associated to *NH_2_OH desorption (Δ*G*_des_) are also provided. The insets illustrate the competition between the electroreduction and desorption of *NH_2_OH. The most favored species are highlighted in bold. Color code: Cu (gray), Zn (dark blue), O (red), N (blue), H (white).

The computed Gibbs energy diagrams on the different electrocatalysts are depicted in Figure 6a (left). These are reported at the experimental potentials where the same total current density of 100 mA/cm^2^ can be achieved on the Zn, Cu, and Zn_93_Cu_7_ electrocatalysts. These diagrams revealed that the first PCET to form the intermediate *NOH (i.e., hydrogenated at the O, eq 2) is thermodynamically favorable on pure Zn (i.e., −0.95 eV), whereas this process is slightly endergonic both on pure Cu and Zn_93_Cu_7_ (i.e., +0.17 and +0.11 eV, respectively). These findings and the highly exergonic formation of N_2_ predicted on Zn (i.e., – 5.38 eV) are in line with the higher FE toward N_2_ observed in experiments on Zn compared to Cu and Zn_93_Cu_7_ (Figure 4b). Hence, theoretical calculations support hypothesis 4 that the formation of NH_2_OH (which eventually produces oxime) on pure Zn is disadvantaged by the competing NO_3_R pathway toward N_2_, which is marginally accessible on Cu and Zn_93_Cu_7_. These results could also explain the lower yields of oxime achieved on pure Zn relative to the Zn_93_Cu_7_ alloy (Figure 4b).

The production of NH_2_OH and NH_3_ relies instead on the formation of the *NHO intermediate (i.e., hydrogenated at the N), which is thermodynamically more favorable than *NOH on all the electrocatalysts investigated in this work (Figure 6a, right). Notably, while DFT calculations predict the generation of both *NOH and *NHO on Zn(101) to be essentially irreversible at room temperature due to their associated highly exergonic energies (i.e., −0.95 and −1.77 eV, respectively), for Cu and Zn_93_Cu_7_, only the formation of *NHO is thermodynamically driven (i.e., −0.28 and −0.70 eV, respectively). Hence, the relative stability between the *NHO vs *NOH intermediates and the binding strength of NH_2_OH can be used as descriptors to rationalize product selectivity, as we discuss in detail below.

The reduction of *NHO toward *NH_2_OH and NH_3_ was investigated via a series of PCET steps. According to DFT calculations, the lowest energy pathway for *NH_2_OH formation involves the sequential hydrogenation of the N and O atoms, as outlined in eqs 5 and 6.
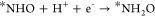
5
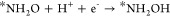
6

As seen from Figure 6a (right), both processes are exergonic on Zn, Cu, and Zn_93_Cu_7_, supporting the formation of the *NH_2_OH intermediate on all these electrocatalysts. In particular, the trend obtained from calculations, from more to less exergonic, is Zn > Zn_93_Cu_7_ > Cu.

The subsequent electroreduction of *NH_2_OH to NH_3_ was then examined according to the mechanism shown in eqs 7–9.
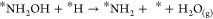
7
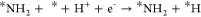
8
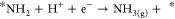
9

Again, calculations predict the electroreduction of *NH_2_OH to NH_3_ to be thermodynamically downhill, in line with the experimental observation of NH_3_ with all the studied electrocatalysts (Figures 4b and 5a). However, we note that *NH_2_OH electroreduction is competing with *NH_2_OH desorption from the electrode surface, which is followed by a chemical reaction with the cyclohexanone in solution to produce oxime. Therefore, to shed light on the different NH_3_/oxime selectivities observed in experiments, we set out to compute both the thermodynamic driving force for *NH_2_OH desorption (Δ*G*_des_) and the kinetic barrier for *NH_2_OH electroreduction (Δ*G*^‡^), the results of which are summarized in Figure 6b. Notably, the *NH_2_OH desorption is almost thermoneutral on Cu (−0.03 eV), slightly endergonic on Zn (+0.19 eV), and exergonic on the Zn_93_Cu_7_ alloy (−0.14 and −0.44 eV from Zn and Cu sites, respectively). For the modeling of the *NH_2_OH electroreduction, we note that this step involves the hydrogenation of the O atom with the concomitant cleavage of the N–O bond, which can be promoted by either the aqueous electrolyte or the H surface coverage. However, given that the p*K*_b_ of NH_2_OH in water is ca. 8.1,^58^ it is unlikely that NH_2_OH will be protonated by the aqueous electrolyte at the experimental pH 7. Hence, we focused on modeling the reaction kinetics of the *NH_2_OH electroreduction by sourcing a H atom from the electrode surface.^56^ Furthermore, because of the relatively large distance between the O lone pairs in *NH_2_OH and the nearest surface H (ca. 3.0 Å on Cu(111) and ca. 4.2 Å on both Zn_93_Cu_7_(101) and Zn(101)), we envisioned this process to be promoted by the aqueous electrolyte. To capture this in our kinetic studies, we introduced two H_2_O molecules to mediate the H transfer. This led to the following energy barriers for the different electrocatalysts: Cu (1.11 eV) ∼ Zn_93_Cu_7_ (1.05 eV) < Zn (1.66 eV) (Figure 6b). With these values of Δ*G*^‡^ and Δ*G*_des_, we can now rationalize the NH_3_/oxime selectivities on Cu, Zn_93_Cu_7_, and Zn as follows. On pure Cu, *NH_2_OH electroreduction (with moderate Δ*G*^‡^) is favored over *NH_2_OH desorption, rendering NH_3_ as the major NO_3_R product. On the other hand, the high Δ*G*^‡^ found for pure Zn hinders *NH_2_OH electroreduction, while the slightly endergonic Δ*G*_des_ allows *NH_2_OH desorption at room temperature, coherently with the product distribution observed experimentally (Figures 4b and 5a). Finally, on the Zn_93_Cu_7_ alloy, the moderate Δ*G*^‡^ and exergonic Δ*G*_des_ allow both the electroreduction and desorption of *NH_2_OH to occur at room temperature, in agreement with the formation of oxime and NH_3_ as major products (Figures 4b).

## Conclusions

In conclusion, we have prepared a Zn_93_Cu_7_ alloy that can drive the one-pot electrosynthesis of cyclohexanone oxime through NO_3_R with a yield of 97% and FE of 27% at 100 mA/cm^2^. This electrochemical method not only enables the production of this important chemical precursor under mild conditions (room temperature, atmospheric pressure, aqueous solution, neutral pH, no toxic/expensive catalysts) compared to conventional chemical routes but also offers a promising strategy to upgrade nitrate into value-added organonitrogen compounds. In addition, this work demonstrates the central role of the *NH_2_OH intermediate in this tandem EChem–Chem process, as well as the importance of controlling the surface adsorption of nitrogen species in electrosynthesis. The distinct behaviors of Zn, Cu, and ZnCu alloys observed in the experimental and theoretical studies reported in this work reveal that Cu-rich electrocatalysts can drive the NO_3_R at lower overpotentials to due to their strong affinity for NO_3_^–^ but favor further electroreduction of *NH_2_OH into NH_3_. In contrast, Zn-rich electrocatalysts require higher overpotentials for NO_3_R but facilitate the chemical reaction between *NH_2_OH and cyclohexanone to produce oxime. Overall, this work demonstrates a novel electrochemical strategy to upgrade nitrate and drive the sustainable synthesis of cyclohexanone oxime. This study also highlights the potential and key aspects for the development of EChem–Chem tandem processes for the electrification of industrial syntheses.

## Supporting Information Available

Experimental and computational methods, compositions of different Zn/Cu catalysts, computed Δ*G*_*nX*_ values, H-cell setup, chronopotentiometry graph, mass spec, NMR, HPLC and IC analysis of the products, SEM, EDX, ICP, ECSA, XRD, and XPS characterizations of the catalysts, reusability of the electrocatalyst, detection of NH_2_OH, nitrate reduction without cyclohexanone, and computational details for the surface coverage of electrocatalysts (PDF)
