# Supplementary material for: Sustainable Electrosynthesis of Cyclohexanone Oxime through Nitrate Reduction on a Zn–Cu Alloy Catalyst
Source: ACS Catal. 2024 Feb 15;14(5):3287–97. doi: 10.1021/acscatal.3c05388 (PMC10913030; doi:10.1021/acscatal.3c05388)
Supplement: Supplementary file 1 — cs3c05388_si_001.pdf [file cs3c05388_si_001.pdf]

Supplementary Information

Sustainable Electrosynthesis of Cyclohexanone Oxime through Nitrate Reduction on a Zn-Cu Alloy Catalyst

Jonathan Sharp[1†], Anna Ciotti[2†], Hayley Andrews[1], Shaktiswaran R. Udayasurian[1], Max García-Melchor[2*], Tengfei Li[1*]

[1]School of Chemistry and Environment, Manchester Metropolitan University, Chester Street, Manchester, M1 5GD, United Kingdom.

[2]School of Chemistry, CRANN and AMBER Research Centres, Trinity College Dublin, College Green, Dublin 2, Ireland.

*Correspondence should be addressed to: garciamm@tcd.ie ; t.li@mmu.ac.uk

†These authors have equal contributions to this work.

Table of Contents

Experimental methods 2

Computational details........ 4

Supplementary tables 8

Figure S1. Electrochemical H-cell setup........ 10

Figure S2. Chronopotentiometry graph 10

Figure S3. Identification of cyclohexanone oxime ($C_6H_{11}NO$) by mass spectroscopy 11

Figure S4. HPLC results 12

Figure S5. IC analysis........ 13

Figure S6. SEM images for the $Zn_{93}Cu_7$ electrocatalyst 13

Figure S7. XPS analysis for the stability test of $Zn_{93}Cu_7$ electrocatalyst 14

Figure S8. Reusability of $Zn_{93}Cu_7$ electrocatalyst........ 15

Figure S9. Isotopic labelling experiments........ 15

Figure S10. XPS results........ 16

Figure S11. SEM images for different electrocatalysts 16

Figure S12. XRD results 17

Figure S13. EDX analysis for the compositions of different Zn-Cu alloys 17

Figure S14. Measurements of the electrochemical active surface area........ 18

Figure S15. Detection of NH_2OH........ 19

Figure S16. Electrochemical nitrate reduction on $Zn_{93}Cu_7$ catalyst without cyclohexanone. 19

Figure S17. Computational details for the surface coverage of electrocatalysts........ 20

References 21

Supplementary Information

Experimental methods

Electrocatalyst preparation

The Zn/Cu catalysts were prepared by DHBT electrodeposition. Copper foil (0.127mm, 99.9%, Alfa Aesar) was sonicated in deionized (DI) water and acetone for 15 min each and covered by Teflon tape to leave an exposed area of 1×1 cm^2. The Cu foil was immersed in 100 mL aqueous electrolyte containing 1.5 M H_2SO_4 and 0.2 M metal precursors. Different ratios of $ZnSO_4{\cdot}7H_2O$ (99%, Alfa Aesar) and $CuSO_4{\cdot}5H_2O$ (99%, Alfa Aesar) were used to prepare a series of Zn-Cu alloys with different compositions. Recipes of Zn/Cu ratio in precursor solution and corresponding catalyst compositions can be found in Table S1. Galvanostatic electrodeposition was performed at a constant current of 3 A for 60 s in a two-electrode system where Cu foil served as cathode and a Pt mesh (99.9%, Sigma-Aldrich) served as anode.

Physical characterizations

SEM images were obtained using a Supra 40VP SEM (Carl Zeiss Ltd., Cambridge, UK) fitted with a field emission electron gun. Samples were mounted onto aluminium SEM pin stubs (12 mm diameter, Agar Scientific, Essex, UK) using adhesive carbon tabs (12 mm diameter, Agar Scientific, Essex, UK). EDX analysis was performed using an Apollo 40 SDD detector (EDAX Ltd., Tilberg, The Netherlands).

XRD data were collected on a PANalytical X'pert Powder X-ray diffractometer using Cu Kα radiation with generator settings of 45 kV and 40 mA. Data were collected in the 2θ range of 10-140° with a step size of 0.013° and a measuring time of 89 s/step. The samples were rotated at 60 rpm during the data collection.

ICP-OES measurements were carried out with a Thermo Scientific iCAP6300 Duo ICP instrument with a Cetac ASX520 auto sampler. A Thermo K-type concentric glass nebuliser and an unbaffled spray chamber with 2mm injector were employed. The Zn-Cu alloy electrodes were dissolved in HNO_3 solution for ICP analysis (control experiments with bare Cu foils were performed to subtract the background Cu signals). The Zn and Cu were detected at wavelengths of 206 nm and 325 nm, respectively.

XPS data were acquired using an AXIS Supra (Kratos Analytical Ltd., UK), equipped with an Al X-ray source (1486.6 eV). X-rays were monochromated using a 500 mm Rowland circle quartz crystal X-ray mirror. The binding energy was referenced to the C 1 s line at 284.8 eV for calibration. Curve fitting was performed applying a Gaussian function.

The reaction aliquots were analyzed by ^1H-NMR, ^{13}C-NMR and MS to confirm the formation of cyclohexanone oxime. NMR data were collected using a JEOL 500 MHz NMR spectrometer. Deuterium oxide (D_2O) was added to the aqueous reaction mixture as the deuterium solvent. For the detection of NH_3 by ^1H-NHR, the reaction sample was acidified with aqueous 0.1 M H_2SO_4 to convert NH_3 into NH_4^+. MS data were collected on an Agilent 6540 Q-Tof LC-MS (ion source: dual ESI). The peak of cyclohexanone oxime was identified by searching in cation mode, *i.e.* $(M+H)^+$, $(M+K)^+$, etc.

In-situ Raman spectra were collected with a Horiba Scientific XploRA Plus Raman Microscope and an *in-situ* electrochemical cell (Redox.me Ltd). The Zn/Cu electrocatalyst was used as working electrode to keep the plane of the sample perpendicular to the incident laser. A Pt wire and an Ag/AgCl electrode (saturated KCl) were used as counter and reference electrodes, respectively. A 0.1 M KNO_3 solution was used as electrolyte. A 10x objective was

employed to focus on the surface of the electrode and a Raman spectrum was recorded during the electrochemical experiment. Measurements were made using a 638 nm laser, with an acquisition time of 5 s and two accumulations.

Electrochemical measurements

A Metrohm Autolab PGSTAT100N potentiostat and a gas-tight H-type electrochemical cell with a Nafion proton exchange membrane (Fuel Cell Store) as the separator were used for all electrochemistry experiments. The electrocatalyst (surface area = 1 cm^2) was used as working electrode. The cathode compartment contained 16 mL of 0.5 M aqueous KPi (KH_2PO_4, 99%, Alfa Aesar, and KOH, 99%, Alfa Aesar) buffer solution (pH 7.0) with 100 mM KNO_3 (99%, Alfa Aesar) and 25 mM cyclohexanone (99%, Alfa Aesar). The solution was purged with Ar gas for 30 min before electrochemical measurements. For isotopic labelling experiments, KNO_3 was replaced by $K^{15}NO_3$ (98%, Sigma-Aldrich). Pt was used as the counter electrode in the anode compartment, which also contained 0.5 M KPi aqueous buffer solution (pH 7.0) and 0.1 M KNO_3. A photo of the H-cell setup can be found in **Figure S1**.

The electrosynthesis of cyclohexanone was performed at constant current densities and the reaction time was adjusted to keep a constant value of charges. For example, the electrochemical reactions at 100 mA/cm^2 and 50 mA/cm^2 were performed for 9,000 s (2.5 h) and 18,000 s (5 h) to keep a constant value of charges (900 C). All potentials were measured against an Ag/AgCl reference electrode (saturated KCl) and converted to the RHE reference scale using Eq. S1:

$$E(V_{RHE}) = E_{Ag|AgCl} + 0.197 + 0.059 \times pH \qquad (S1)$$

Product quantifications

The concentrations of cyclohexanone and cyclohexanone oxime were quantified by an Agilent HPLC equipped with a Waters C8 column (4.6 × 250 mm) and a UV-Vis detector. Cyclohexanone was detected at a wavelength of 280 nm, while cyclohexanone oxime was detected at 220 nm. Water and acetonitrile were used as the eluents. The reaction mixture (125 μL) was diluted by deionized water (375 μL) and an aqueous 20 mM benzoic acid solution (100 uL) was added as the internal standard for each HPLC measurement. A series of standard solutions with known concentrations of cyclohexanone and cyclohexanone oxime were prepared to plot a linear calibration curve.

The concentration of NO_3^-, NO_2^-, and NH_3 were analyzed by a Thermo Scientific IC (ICS5000+). The anion detection utilizes an IonPac AG18 guard and an AS18 separation column (2 mm) with a KOH eluent gradient of 18 mM at the beginning of chromatogram, which increased to 50mM after 16 min. The cation detection utilizes an IonPac CG16 guard and a CS16 separation column (3 mm) with a 39 mM methanesulfonic acid eluent (which can acidify NH_3 into NH_4^+). The IC peaks were identified and quantified by comparing the peaks to a series of standard solutions with known concentrations of each species.

The gas products were analyzed by injecting 50 μL of gas from the headspace of the cathode compartment into an Agilent 8890 GC equipped with a MolSieve 5A (2.44 m × 2 mm ID, Agilent) column. H_2 and N_2 gases were detected by a thermal conductivity detector (TCD) using He as the carrier gas. Calibration curves for H_2 and N_2 were created by plotting the peak area versus concentration in standard calibration gas.

The concentration of NH_2OH was analyzed according to a colorimetric method reported elsewhere, where Fe^{3+} was reduced by NH_2OH to Fe^{2+} and formed an orange complex with

1,10-phenanthroline which has a UV-Vis absorbance peak at 510 nm.[1,2] The reaction sample (10 μL) was diluted by deionized water (2990 μL), and aqueous acetate buffer (1 M NaAc + 1 M HAc, 100 μL), 4 mM aqueous $NH_4Fe(SO_4)_2$ solution (100 μL) and 10 mM 1,10-phenanthroline in ethanol (100 μL) were sequentially added to the sample. A series of standard solutions with known concentrations of NH_2OH were prepared to plot a linear calibration curve. The samples were measured by an Agilent Cary 60 UV-Vis spectrometer.

The product yield is defined as Eq. S2, using cyclohexanone oxime as an example:

$$Yield\ (cyclohexanone\ oxime) = \frac{concentration\ of\ cylcohexanone\ oxime}{initial\ concentration\ of\ cyclohexanone} \quad (S2)$$

The selectivity of a certain nitrogen product is defined as Eq. S3, using S3, using cyclohexanone oxime as an example:

$$N\ selectivity\ (cyclohexanone\ oxime) = \frac{concentration\ of\ cylcohexanone\ oxime}{initial\ concentration\ of\ NO_3^-} \quad (S3)$$

The Faradaic efficiency towards a certain product (FE_x) is calculated as Eq. S4

$$FE_x = \frac{16\ mL * conc._x \left(\frac{mol}{mL}\right) * n * 96485\ C/mol}{charge\ (C)} * 100\% \quad (S4)$$

Where n is the number of electrons required to make one product molecule from NO3RR (e.g., n=6 for cyclohexanone oxime or NH_2OH).

Computational details

Periodic-DFT calculations were performed with the Vienna Ab Initio Simulation Package (VASP, revision 6.2.0),[3] adopting the Bayesian Error Estimation Functional (BEEF).[4] Core electrons were described through projector augmented wave pseudopotentials,[5] and valence electrons through plane waves with an energy cut-off of 500 eV. The electronic occupancy in molecules was described with a gaussian smearing of width 0.05 eV, while the Methfessel-Paxton smearing[6] with width 0.2 eV was adopted for solid state systems. All calculations were spin polarized. Molecular species were optimized at the Γ-point, while solid state simulations were carried out with a Γ-centered k-point grid of density $\simeq$ 28 points×Å. Geometry relaxations were performed with a conjugate gradient algorithm with a step size of 0.1 Å and convergence criteria of 10^{-6} eV and 0.01 Å/eV for the electronic and ionic steps, respectively. Transition state (TS) structures for the hydrogenation of $*NH_2OH$ to $*NH_2 + H_2O_{(g)}$ were obtained with the improved dimer[7] and climbing-image nudged elastic band (CI-NEB) methods.[8] The nature of all the stationary points was confirmed by frequency calculations, adopting the finite-differences method with step-size of 0.02 Å. Imaginary frequencies lower than 100 cm^{-1} were replaced by real frequencies of 12 cm^{-1}, if present, following the approach adopted by Brogaard *et al.*[9]

Gibbs energy corrections were computed at the experimental temperature of 298 K and 1 atm of pressure, using the Atomic Simulation Environment (ASE)[10] thermochemistry module. Corrections for gas molecules were determined according to the ideal gas model, while the harmonic limit model was adopted for adsorbate species. For these calculations, all the atoms were vibrated in molecular optimizations, and only the adsorbates in surface slabs.

Supplementary Information

All structures were built using ASE. For the optimization of molecular species, a vacuum of 15 Å was applied in all directions to avoid interactions between repeating images. For bulk solids, the cell structure of minimum energy above Hull was downloaded from the Materials Project database.[11] The lattice parameters were varied by 1% five times, and each image was optimized relaxing only the atom positions. The resulting energies and volumes were fitted to the Birch Murnaghan equation of state,[12] and its minimum was then relaxed at constant volume while allowing the cell shape to change, yielding the optimized bulk structure which was later on used to create the surface models. Four-layered Cu(111) and Zn(101) surface slabs were cleaved adding a vacuum space of 15 Å in the direction perpendicular to the surface. The atoms in the two bottom layers were fixed to the bulk positions while the rest of atoms were allowed to relax.

For the $Zn_{93}Cu_7$ alloy, we assumed the aggregation of Cu atoms to be unlikely since the experimental sample was found by XRD to have the least abundant element dispersed in the most abundant one, presumably due to the favorable interactions between Cu and Zn.[13] Moreover, given the similar atomic radii of Cu and Zn, and the low concentration of Cu in $Zn_{93}Cu_7$, we expected this alloy to maintain the same crystal structure as pure Zn. Therefore, a four-layer $Zn_{93}Cu_7$(101) surface slab was constructed by substitutionally doping a *p*(2×2)-Zn(101) surface, resulting in a Cu:Zn ratio of 6.3%, which compares very well with the experimental value of 7%. The substitution of a Zn atom by Cu on a step site was calculated to be more favorable than on a terrace site by 0.09 eV. Therefore, we adopted a *p*(2×2) supercell with a single Cu atom on a step for the NO_3R reactivity studies.

Surface coverage analyses were carried out on Cu(111) and Zn(101), first investigating the binding of a single *H, *OH and *O group in the *fcc*, *hcp*, *top* and *bridge* site of each *p*(2×2) supercell. The coverage density was then increased by progressively populating the most stable sites for each adsorbate, interrupting the analysis when molecular species (*i.e.* H_2, O_2) were evolved and/or surface reconstruction occurred. The explored coverages were then labelled, indicating the adsorbate type and their density. To express the latter, we noted that every adsorbate increased the population of a specific type of sites (*i.e*. *bridge*, *fcc*, *hcp* or *top*) by 25%, since the slabs presented four sites of each type. Therefore, we decided to multiply the number of occupied sites in the *p*(2×2) slab by 0.25 (*e.g.*, 0.25 H identifies the filling of one site in the *p*(2×2) slab with H, while 1.25 H identifies the filling of five sites in the *p*(2×2) slab with H).

Relative Gibbs energies of the different adsorbed X species (ΔG_{nX}) were computed adopting the computational hydrogen electrode (CHE) model,[14] according to:

$$\Delta G_{nH}(0V_{RHE}) = G_{nH} - \left(G_* + \frac{n}{2}G_{H_2}\right) \quad (S5)$$

$$\Delta G_{nOH}(0V_{RHE}) = G_{nOH} + \frac{n}{2}G_{H_2} - \left(G_* + nG_{H_2O}\right) \quad (S6)$$

Where G_{nX} is the Gibbs energy of the *p*(2×2) supercell bearing n adsorbed X species, G_* is the Gibbs energy of the bare *p*(2×2) supercell, G_{H_2} is the Gibbs energy of a H_2 gas molecule, e is the electron charge, and G_{H_2O} is the Gibbs energy of a H_2O gas molecule. The computed ΔG_X values using Eq. S2-S4 are summarized in **Table S2**.

Supplementary Information

The surface coverage diagrams for pure Zn and Cu show in **Figure S14a** were constructed by plotting versus the applied potential, using the following equations, where E_{RHE} is the applied potential versus RHE:

$$\Delta G_{nH}(E_{RHE}) = \Delta G_{nH}(0V_{RHE}) + neE_{RHE} \qquad (S7)$$

$$\Delta G_{nOH}(E_{RHE}) = \Delta G_{nOH}(0V_{RHE}) - neE_{RHE} \qquad (S8)$$

$$\Delta G_{nO}(E_{RHE}) = \Delta G_{nO}(0V_{RHE}) - 2neE_{RHE} \qquad (S9)$$

For a more detailed description of the construction of these diagrams, we refer the reader to our recent opinion paper.[15]

According to **Figure S14a,** the Cu(111) slab features 75% of the *fcc* sites covered with H atoms. While the surface studies presented here were conducted on uniform coverages, H is known to diffuse at room temperature on transition metals.[15, 16] Additionally, the arrangement of the H atoms can affect reaction energetics.[17] Thus, we deemed it necessary to determine the H distribution that best stabilized the NO_3R intermediates, adopting the adsorption of *NH_2OH as a case example. While NH_2OH desorption was observed on the Cu(111) slab featuring a uniform H coverage, chemisorption of *NH_2OH was achieved by displacing the H atoms away from the binding site on the latter. This system, depicted in **Figure S14b,** was therefore adopted to model all the other intermediates in NO_3R. On the Zn(101) slab, instead, all the *fcc* sites were covered with H at the experimental conditions (**Figure S14a**). Thus, no H diffusion was considered. Given the low concentration of surface Cu, the resting state of the $Zn_{93}Cu_7$(101) alloy was adopted from the surface coverage diagram for Zn(101), which consists of all the *fcc* sites covered with H atoms at the experimental potential of –0.90 V_{RHE}.

The NO_3R mechanism to N_2, NH_2OH and NH_3 was then modelled. This involves both negatively charged (*e.g.* *NO_3^- and *NO_2^-) and neutral intermediates (*e.g.* NO),[18-20] whose energy cannot be directly compared, due to the application of a background charge in the anionic systems. Some works circumvent this issue through a thermodynamic cycle, relating the energy of neutral models of *NO_3 and *NO_2 to that of the solvated NO_3^- and NO_2^-.[19, 20] However, we observed that *NO_3 and *NO_2 absorbed on metal surfaces form the more stable *$NO_3^-$and *NO_2^-, subtracting an electron from the slab and seen by Bader charge analysis. Thus, their Gibbs adsorption energies (ΔG_{*NO3} and ΔG_{*NO2}) will mainly reflect the work functions of different metals in our system (Cu, $Zn_{93}Cu_7$ and Zn), instead of the actual binding energies of *NO_3^- and *NO_2^-. Therefore, the zero of energies was taken to be NO rather than NO_3^-, as the reduction from NO_3^- to NO is widely understood.[19-22] The reaction energetics (ΔG_{*X}), reported in **Table S3**, were calculated according to the CHE, as follows:

$$\Delta G_{*NHO} = G_{*NHO} - \left(G_* + {}^1\!/_2\,\Delta G_{H_2} + G_{NO}\right) + E_{RHE} \qquad (S10)$$

$$\Delta G_{*NOH} = G_{*NOH} - \left(G_* + {}^1\!/_2\,\Delta G_{H_2} + G_{NO}\right) + E_{RHE} \qquad (S11)$$

$$\Delta G_{*NHOH} = G_{*NHOH} - \left(G_* + \Delta G_{H_2} + G_{NO}\right) + 2E_{RHE} \qquad (S12)$$

$$\Delta G_{*NH_2O} = G_{*NH_2O} - \left(G_* + \Delta G_{H_2} + G_{NO}\right) + 2E_{RHE} \qquad (S13)$$

$$\Delta G_{*NH_2OH} = G_{*NH_2OH} - \left(G_* + {}^3\!/_2\,\Delta G_{H_2} + G_{NO}\right) + 3E_{RHE} \qquad (S14)$$

$$\Delta G_{*NH_2,v} = G_{*NH_2,v} - \left(G_* + {}^3\!/_2\,\Delta G_{H_2} + G_{NO} + G_{corr,H}\right) + 3E_{RHE} \qquad (S15)$$

$$\Delta G_{*NH_2} = G_{*NH_2} - \left(G_* + 2\Delta G_{H_2} + G_{NO}\right) + 4E_{RHE} \qquad (S16)$$

Due to the large number of combinations of binding sites (*i.e. bridge, fcc, hcp,* and *top*) and binding modes (*i.e*. mono- and bidentate through the N and O atoms), the following assumptions were made, whenever possible:

i) The coverage analysis showed the most favored site for *O to be the *fcc* on Cu(111) and the *bridge* on Zn(101) (**Table S2**). Therefore, the binding of the intermediates featuring a non-hydrogenated O (*i.e*. *NO, *NHO, $*NH_2O$) was limited to the *fcc* sites for Cu(111), and to the *bridge* sites for Zn(101).

ii) The Lewis structures of the intermediates featuring an -OH group (*i.e*. *NOH and *NHOH) present a radical character on the N atom. Therefore, only monodentate modes through the N and bidentate modes were explored.

iii) ΔG values for the *hcp* sites are not reported in **Table S3**. In fact, the coverage analysis of Cu(111) showed similar energetics for *hcp* and *fcc* sites, with a preference for the *fcc* (**Table S2**), while binding of the reaction intermediates at *hcp* sites on Zn(101) resulted in major surface reconstruction.

iv) Since *fcc* sites on the Zn(101) and $Zn_{93}Cu_7$(101) resting states are fully occupied with H atoms, these sites were deemed unavailable for the binding of reaction intermediates.

v) For the $Zn_{93}Cu_7$ alloy, only the most stable intermediates determined on Zn(101) were modelled (*i.e*. *NO, *NHO, $*NH_2O$, $*NH_2OH$, and $*NH_2$) in the preferred binding mode, with the exception of *NOH due to its crucial role in NH_2OH/NH_3 selectivity.

Computational details, including cartesian coordinates and energies of the optimized structures, are openly accessible in the following ioChem-BD dataset: https://iochem-bd.bsc.es/browse/review-collection/100/313615/e30222361dab058a66569658.

Supplementary Information

Supplementary tables

Table S1. Compositions of Zn-Cu alloys prepared by electrodeposition of Zn^{2+} and Cu^{2+} with different concentrations in the precursor solution. The catalyst composition was analyzed by EDX and ICP. We use the surface composition revealed by EDX to describe the catalyst.

Entry	$ZnSO_4$	$CuSO_4$	Zn/Cu ratio in precursor	Catalyst surface composition (revealed by EDX)	Catalyst bulk composition (revealed by ICP)
1	0.2 M	-	-	Zn	Zn
2	0.2 M	0.0033 M	60	$Zn_{93}Cu_7$	$Zn_{91}Cu_9$
3	0.2 M	0.01 M	20	$Zn_{85}Cu_{15}$	$Zn_{81}Cu_{19}$
4	0.171 M	0.0285 M	6	$Zn_{74}Cu_{26}$	$Zn_{62}Cu_{38}$
5	0.1 M	0.1 M	1	Zn_6Cu_{94}	Zn_3Cu_{97}
6	-	0.2 M	-	Cu	Cu

Table S2. Computed ΔG_{nX} values, in eV, for *H, *OH and *O species on the Cu(111) and Zn(101) *p*(2×2) supercells at 0 V_{RHE} for all the investigated binding sites (*i.e. bridge*, *fcc*, *hcp* and *top*) and coverage densities, as explained in the **Computation details** section. Whenever migration to different sites occurred, these are indicated in place of the ΔG_{nX} values.

%	Site	*H		*OH		*O	
		Cu	Zn	Cu	Zn	Cu	Zn
0.25	*bridge*	*fcc*	0.62	*fcc*	–0.12	fcc	–0.12
	fcc	0.20	0.42	0.16	*bridge*	0.65	*bridge*
	hcp	0.23	*fcc*	0.20	*bridge*	0.77	*bridge*
	top	*fcc*	*fcc*	*hcp*	*bridge*	hcp	*bridge*
0.50	*fcc*	0.42	0.83	0.80	–0.14*	2.76	-
0.75	*fcc*	0.72	1.29	2.68	-	6.03	-
1.00	*fcc*	1.22	1.81	4.65	-	9.83	-
1.25	*fcc* + *hcp*	2.80	-	-	-	-	-

*For the *OH and *O adsorption on Zn(101), the bridge site was determined to furnish the most stable adsorption. Therefore, the 0.50 OH and O coverages were modelled by binding *OH and *O on bridge sites. The 0.50 O covered is not reported as it led to major surface reconstruction.

Table S3. Computed ΔG_{*X} values, in eV, for the reaction intermediates in *NO reduction to NH_2OH and NH_3, at experimental potential, for all the investigated binding modes, as explained in the **Computation details** section. Those that were not expected to be favored based on the Lewis structure of the intermediates are labelled as ‘not favored’. Where the binding resulted in migration to a different site, desorption, or major surface reconstruction, this is indicated in place of the ΔG_{*X} values.

Intermediate	Binding	Cu	$Zn_{93}Cu_7$	Zn
***NO**	N_{bridge}	fcc	-	desorption
	N_{fcc}	–0.01	-	desorption
	N_{top}	–0.02	Zn: 0.17 Cu: 0.42	0.20
	O	desorption	-	desorption
	bi	top	-	top
***NHO**	N_{bridge}	not favored	-	top
	N_{fcc}	top	*H	*H
	N_{top}	–0.28	-	–1.07
	O	0.18	-	reconstruction
	bi	top	–0.70	–1.77
***NOH**	N_{bridge}	fcc	0.18	–0.95
	N_{fcc}	0.17	*H	*H
	N_{top}	0.66	Zn: 0.11	–0.16
	O	not favored	not favored	not favored
	bi	top	desorption	–0.39
***NH_2O**	N_{bridge}	not favored	-	not favored
	N_{fcc}	not favored	*H	*H
	N_{top}	bi	-	–2.08
	O	–0.88	-	–3.34
	bi	–1.06	–1.85	–3.53
***NHOH**	N_{bridge}	–0.82	-	–3.10
	N_{fcc}	–0.43	*H	*H
	N_{top}	bi	-	bi
	O	not favored	not favored	not favored
	bi	–0.81	-	–2.78
***NH_2OH**	N_{bridge}	desorption	-	desorption
	N_{fcc}	desorption	*H	*H
	N_{top}	–1.91	Zn: –3.30 Cu: –3.00	–4.53
	bi	desorption	-	desorption
***$NH_{2,v}$**	N_{bridge}	–3.76	–5.36	–7.28
	N_{fcc}	bridge	*H	*H
	N_{top}	bridge	-	–6.32
***NH_2**	N_{bridge}	–3.75	–5.77	–8.33
	N_{fcc}	not favored	*H	*H
	N_{top}	not favored	-	not favored

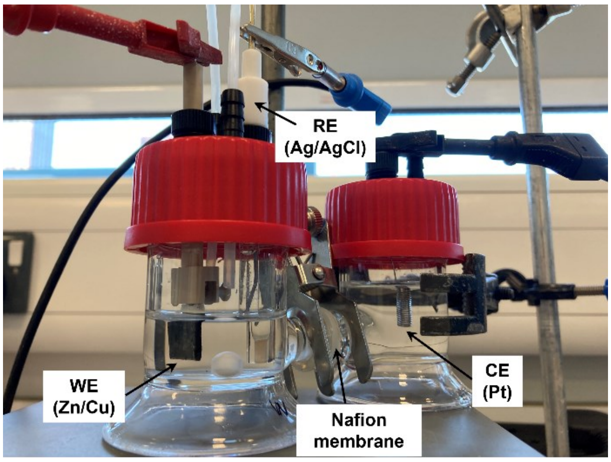

Figure S1. Electrochemical H-cell setup. The Zn/Cu electrocatalyst was used as working electrode. Pt was used as counter electrode. Ag/AgCl was used as reference electrode. A Nafion membrane was used to separate the two compartments of the H-cell.

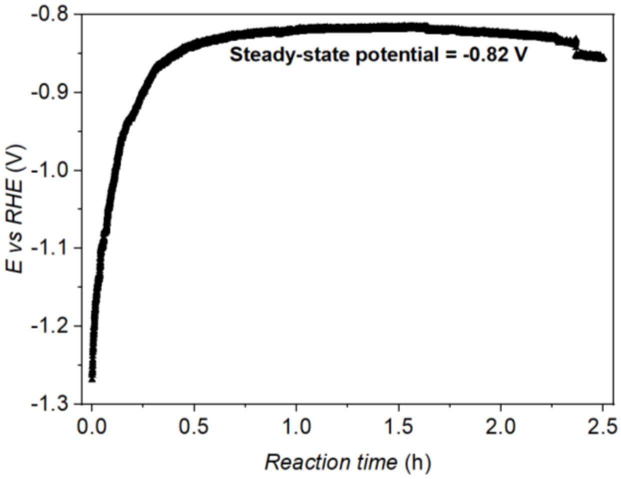

Figure S2. Chronopotentiometry graph for electrochemical nitrate reduction at 100 mA/cm^2 over 2.5 h. Reaction conditions: $Zn_{93}Cu_7$ cathode (surface area = 1 cm^2) immersed in 16 mL aqueous buffer solution (0.5 M KPi, pH 7.0) containing 100 mM KNO_3 and 25 mM cyclohexanone.

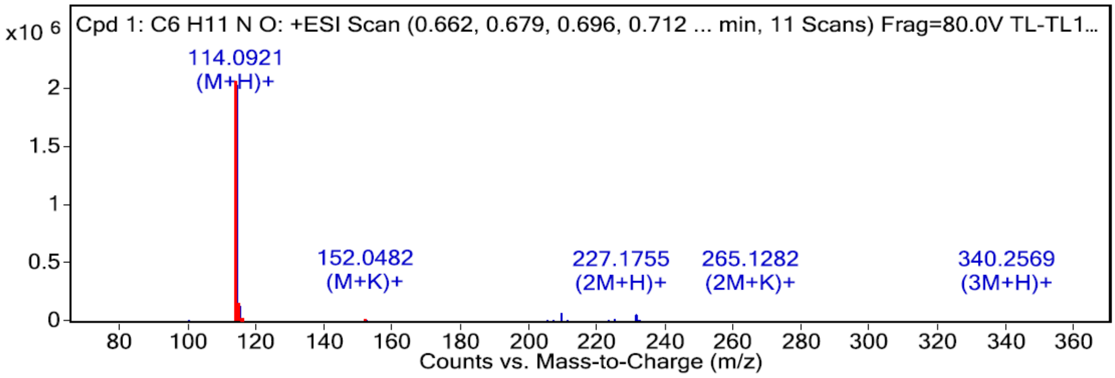

MS Spectrum Peak List

m/z	*Calc m/z*	Diff(ppm)	z	Abund	Formula	Ion
96.0814	96.0808	6.88	1	5434.92	C6H10N	(M+H)+[-H2O]
114.0921	114.0913	6.38	1	2064493.5	C6H12NO	(M+H)+
152.0482	152.0472	6.41	1	9371.72	C6H11KNO	(M+K)+

Figure S3. Identification of cyclohexanone oxime ($C_6H_{11}NO$) by mass spectroscopy (ion source: dual ESI). The peak of cyclohexanone oxime was identified by searching the possible m/z values of $C_6H_{11}NO$ in cation mode, such as $(M+H)^+$, $(M+H)^+$-H_2O and $(M+K)^+$.

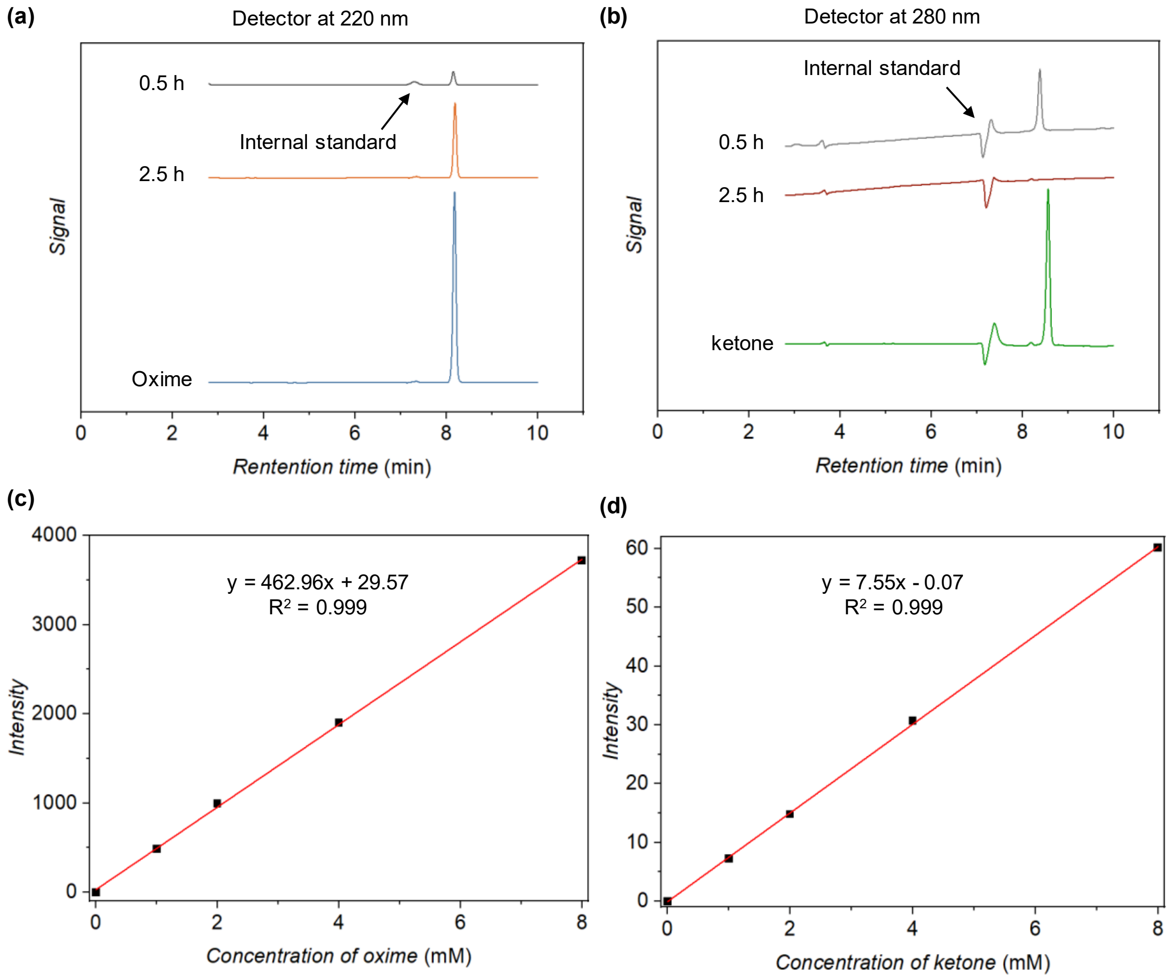

Figure S4. HPLC results. a) Detection of cyclohexanone oxime (UV-Vis detector at 220 nm) in the electrolyte mixture at 0.5 h and 2.5 h, and comparison with pure cyclohexanone oxime. b) Detection of cyclohexanone oxime (UV-Vis detector at 280 nm) in the electrolyte mixture at 0.5 h and 2.5 h, and comparison with pure cyclohexanone. c) Calibration curve for cyclohexanone oxime. d) Calibration curve for cyclohexanone.

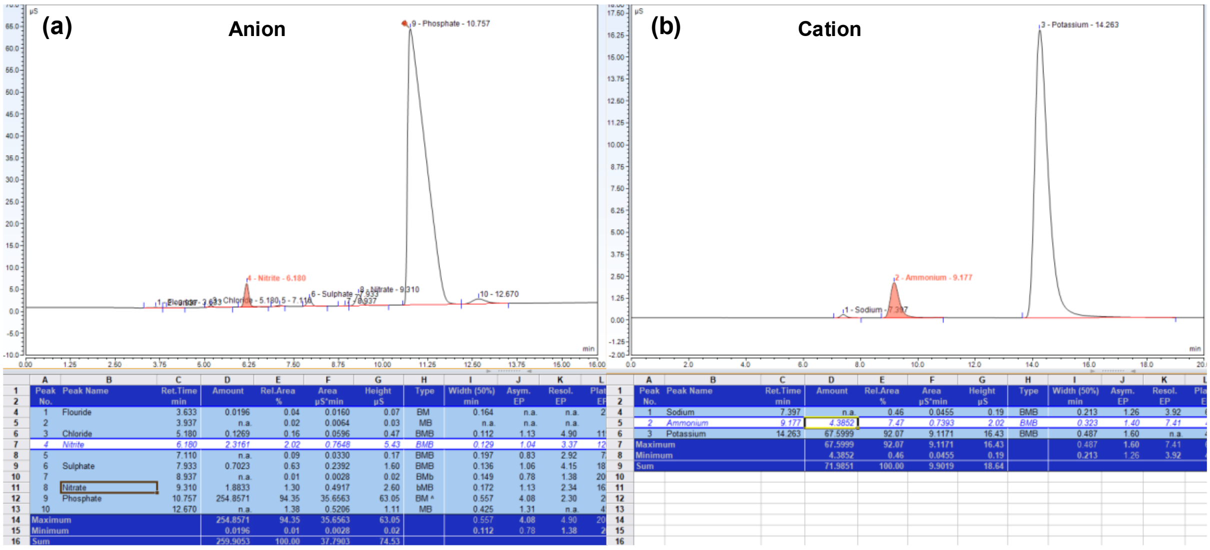

Figure S5. IC analysis of the reaction mixture (diluted 240 times by DI water) for a) anions and b) cations. Nitrite, nitrate and ammonium were detected and quantified by comparing the peaks to a series of standard solutions with known concentrations of each species.

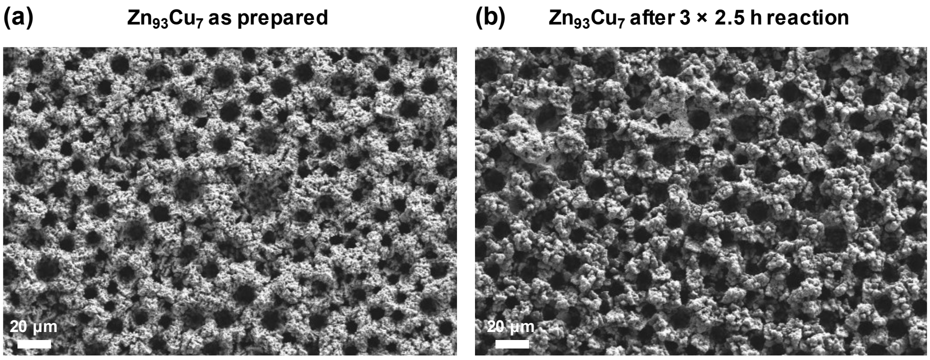

Figure S6. SEM images for the $Zn_{93}Cu_7$ electrocatalyst as prepared (a) and after 3 runs of electrochemical reactions (b). Each run is 2.5 h at 100 mA/cm^2.

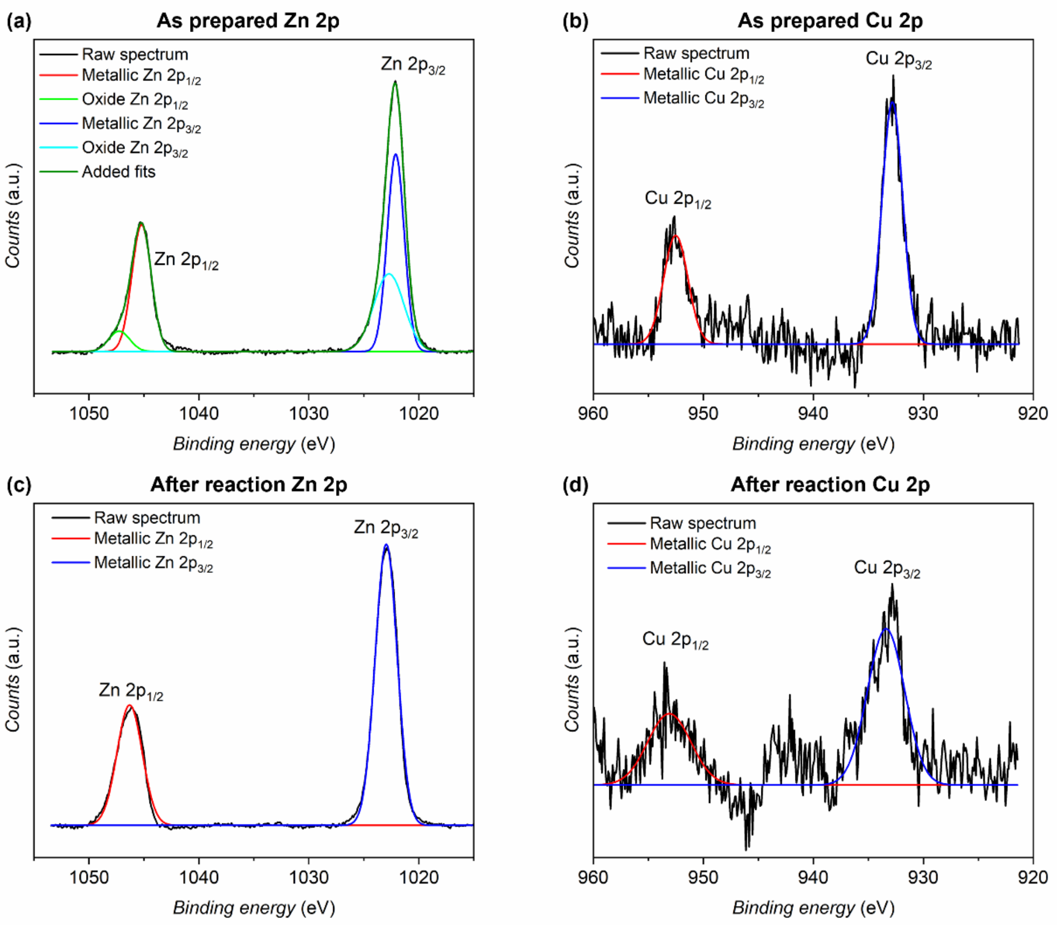

Figure S7. XPS analysis for the stability test of $Zn_{93}Cu_7$ electrocatalyst as prepared (a and b) and after 3 runs of electrochemical reactions (b). Each run is 2.5 h at 100 mA/cm^2.

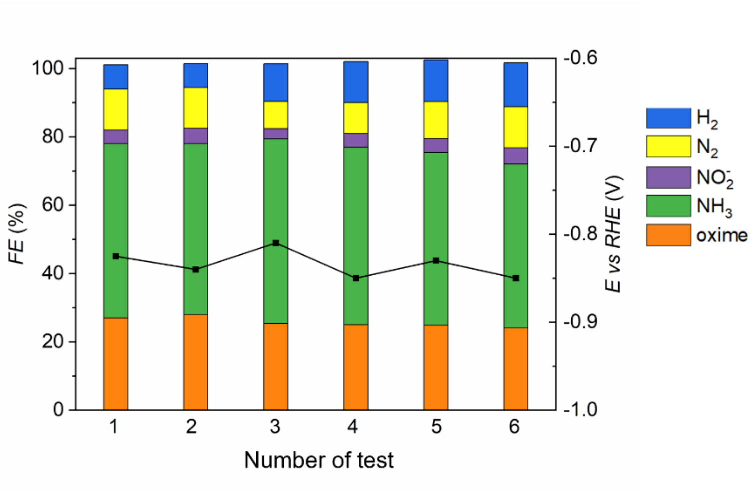

Figure S8. Reusability of $Zn_{93}Cu_7$ electrocatalyst. F.E. and potential required to achieve J_{total} at 100 mA/cm^2 for nitrate reduction. A $Zn_{93}Cu_7$ electrocatalyst was tested for 6 runs of electrochemical reactions (each run is 2.5 h at 100 mA/cm^2).

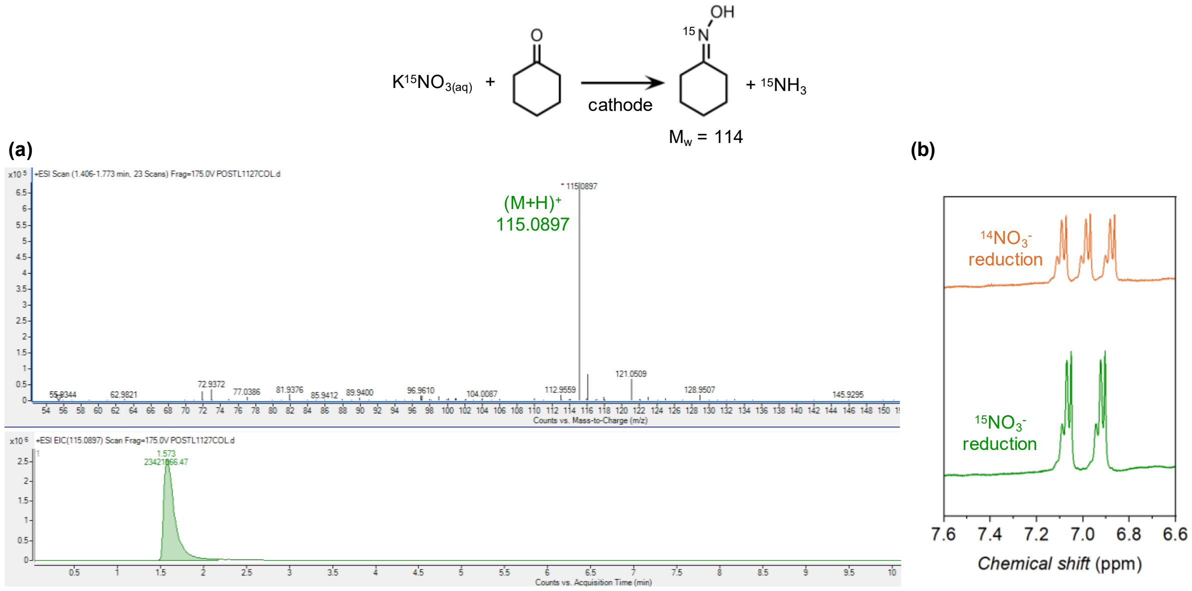

Figure S9. Isotopic labelling experiments. Electrochemical reduction of $K^{15}NO_3$ into ^{15}N-lablled cyclohexanone oxime and $^{15}NH_3$. a) LC-MS Identification of ^{15}N-lablled cyclohexanone oxime (M_w = 114). The peak of cyclohexanone oxime was identified by searching the possible m/z values of $C_6H_{11}{}^{15}NO$ in cation mode, such as $(M+H)^+$. b) ^{1}H NMR spectra for $^{14}NO_3^-$ reduction ($^{14}NH_4^+$ as the product) and $^{15}NO_3^-$ reduction ($^{15}NH_4^+$ as the product).

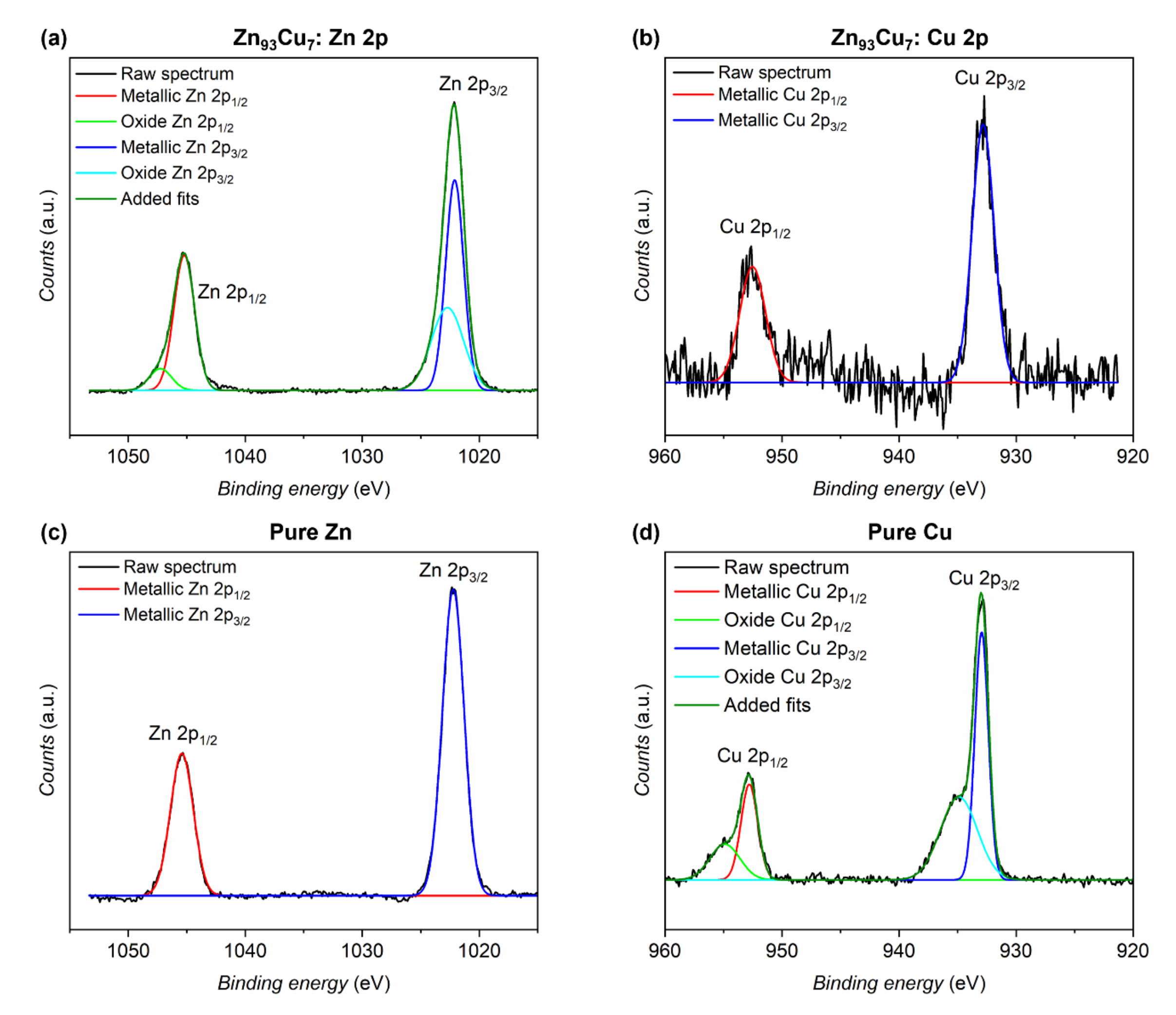

Figure S10. XPS results for $Zn_{93}Cu_7$ alloy (a and b), pure Zn (c) and pure Cu (d).

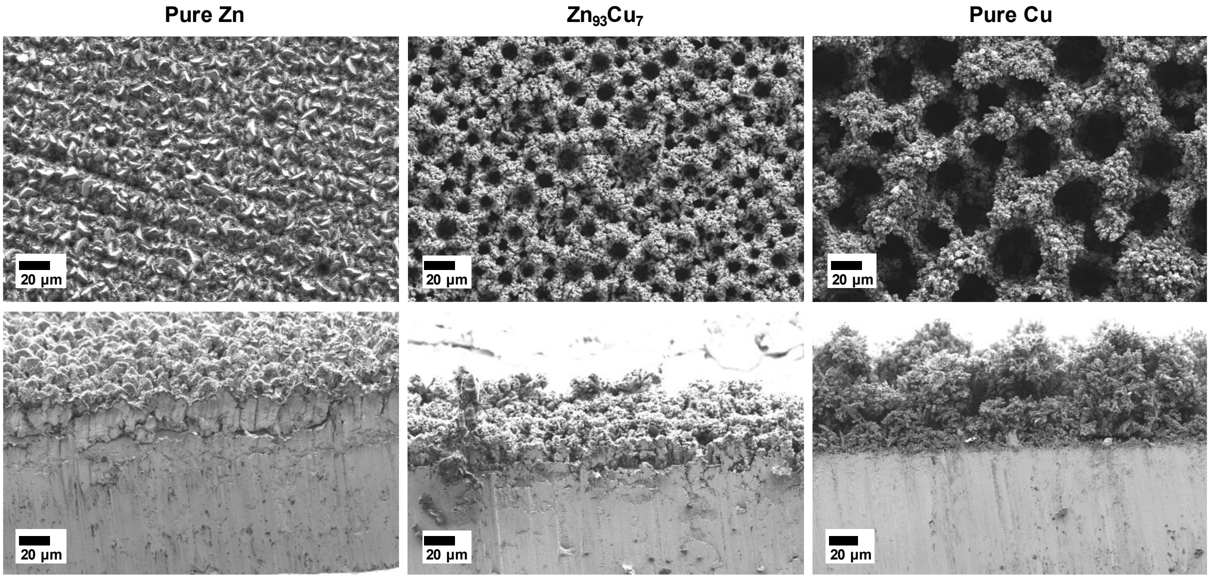

Figure S11. SEM images for different electrocatalysts. Top views (top) and cross-sectional views (bottom) of SEM images for pure Zn, $Zn_{93}Cu_7$ alloy and pure Cu.

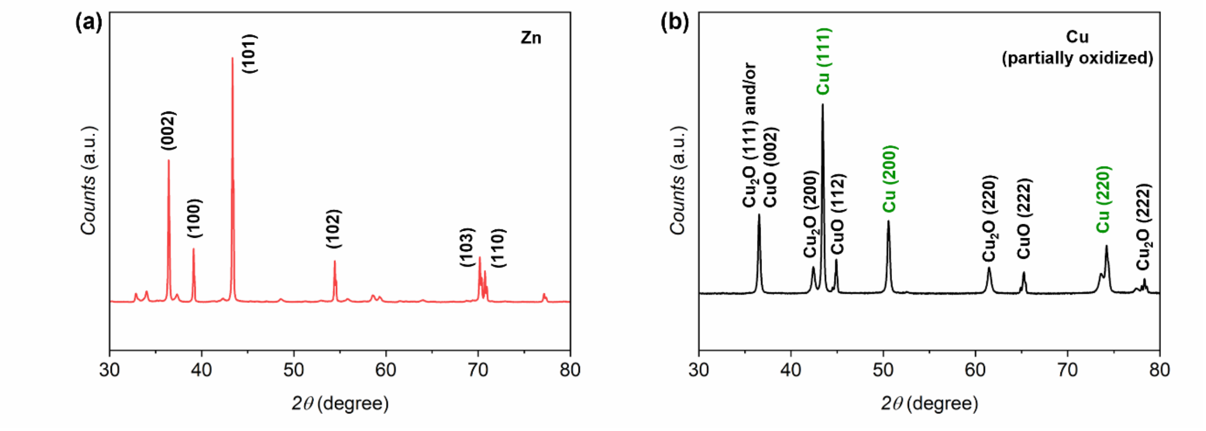

Figure S12. XRD results for pure Zn (a) and pure Cu (b). The Cu is partially oxidized to Cu_2O and CuO.

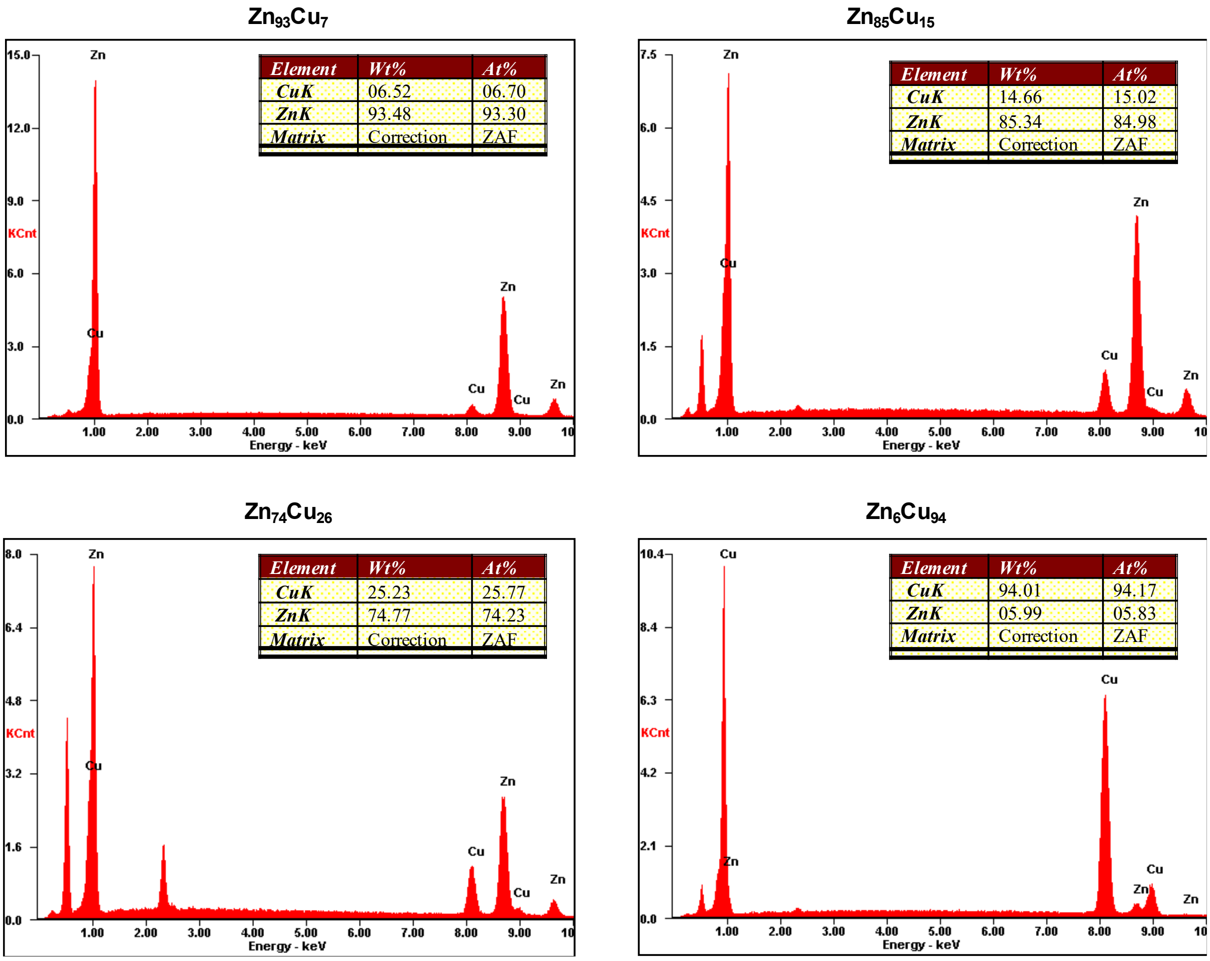

Figure S13. EDX analysis for the compositions of different Zn-Cu alloys.

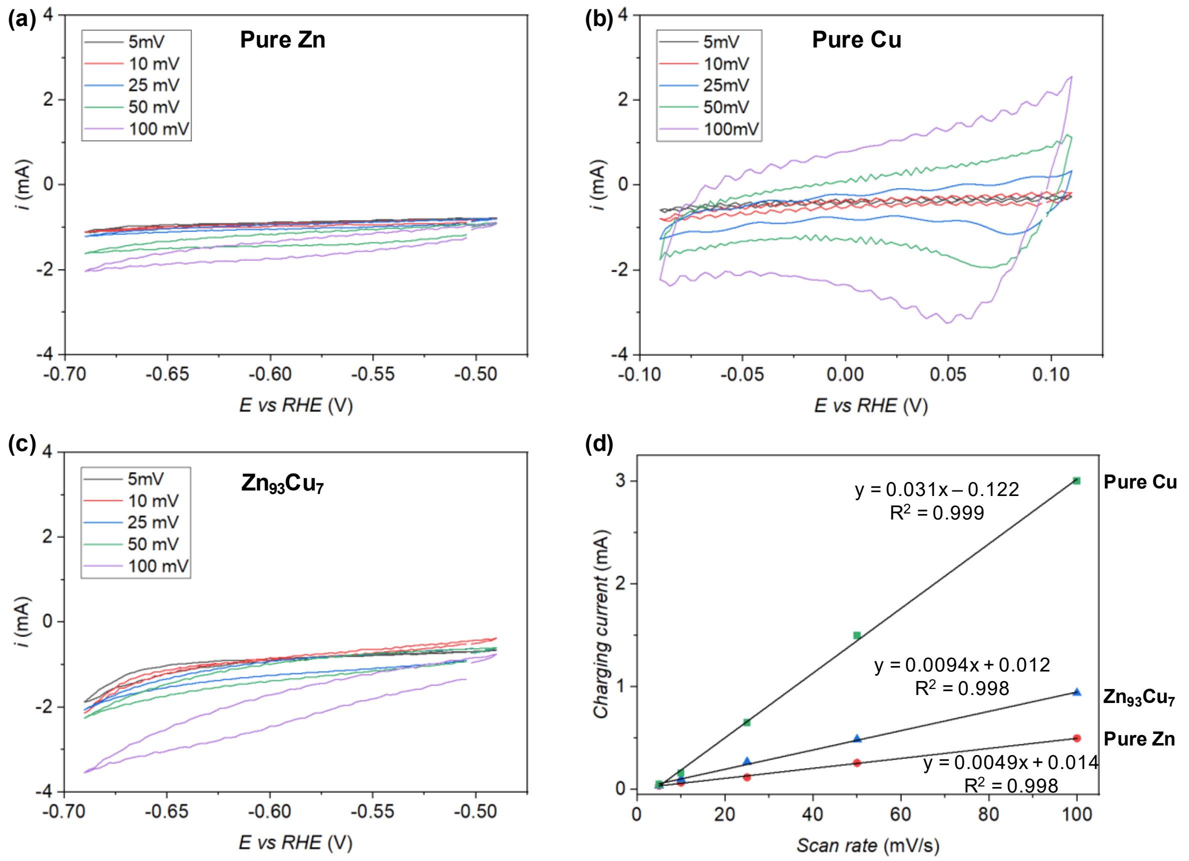

Figure S14. Measurements of the electrochemical active surface area (ECSA) for pure Zn, $Zn_{93}Cu_7$ alloy and pure Cu. Measurement condition: electrocatalyst (surface area = 1 cm^2) was immersed in 16 mL aqueous 0.1 M KCl buffer solution. Pt was used as counter electrode in the anode compartment. Ag/AgCl was used as the reference electrode.

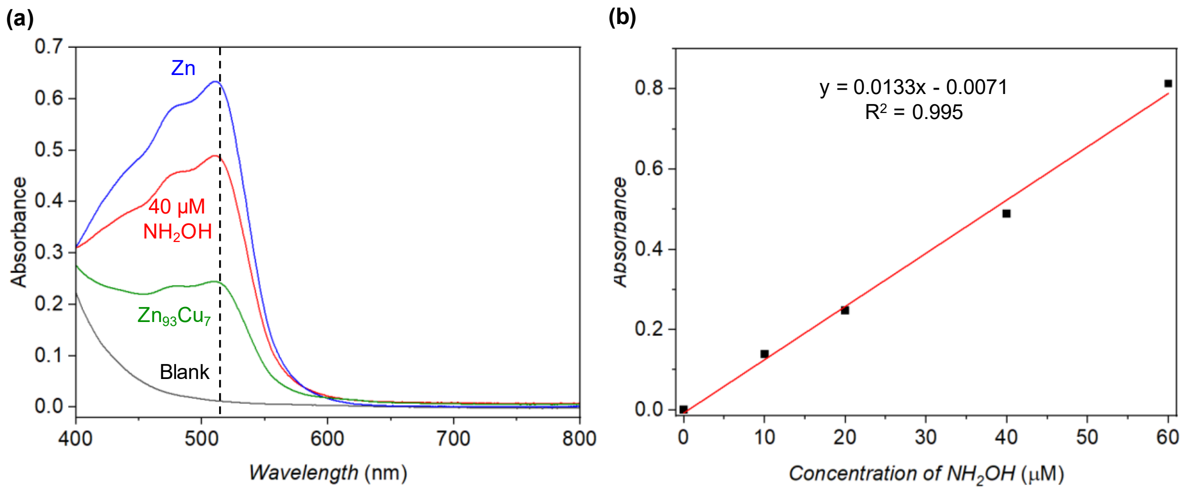

Figure S15. Detection of NH_2OH. a) UV-Vis spectra for the orange complex formed due to NH_2OH. Diluted electrolyte mixtures after 2.5 h reaction catalyzed by pure Zn or $Zn_{93}Cu_7$ were compared with standard NH_2OH solution and blank DI water (treated with the same colorimetric method). b) Calibration curve for NH_2OH using the absorbance at 510 nm.

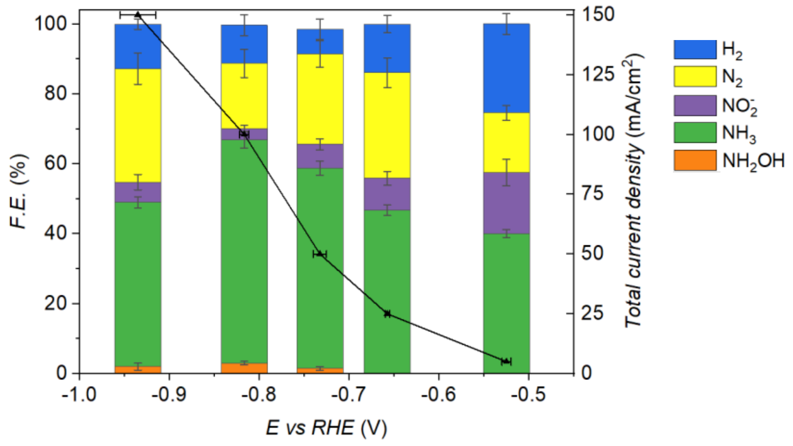

Figure S16. Electrochemical nitrate reduction on $Zn_{93}Cu_7$ catalyst without cyclohexanone**.** F.E. and potential required to achieve 100 mA/cm^2 are plotted again potential. Reaction conditions: $Zn_{93}Cu_7$ cathode (surface area = 1 cm^2) immersed in 16 mL aqueous buffer solution (0.5 M KPi, pH 7.0) containing 100 mM KNO_3. Potential values were obtained from the steady-state potentials for constant current electrolysis. Error bars correspond to the standard deviation of triplicate experiments.

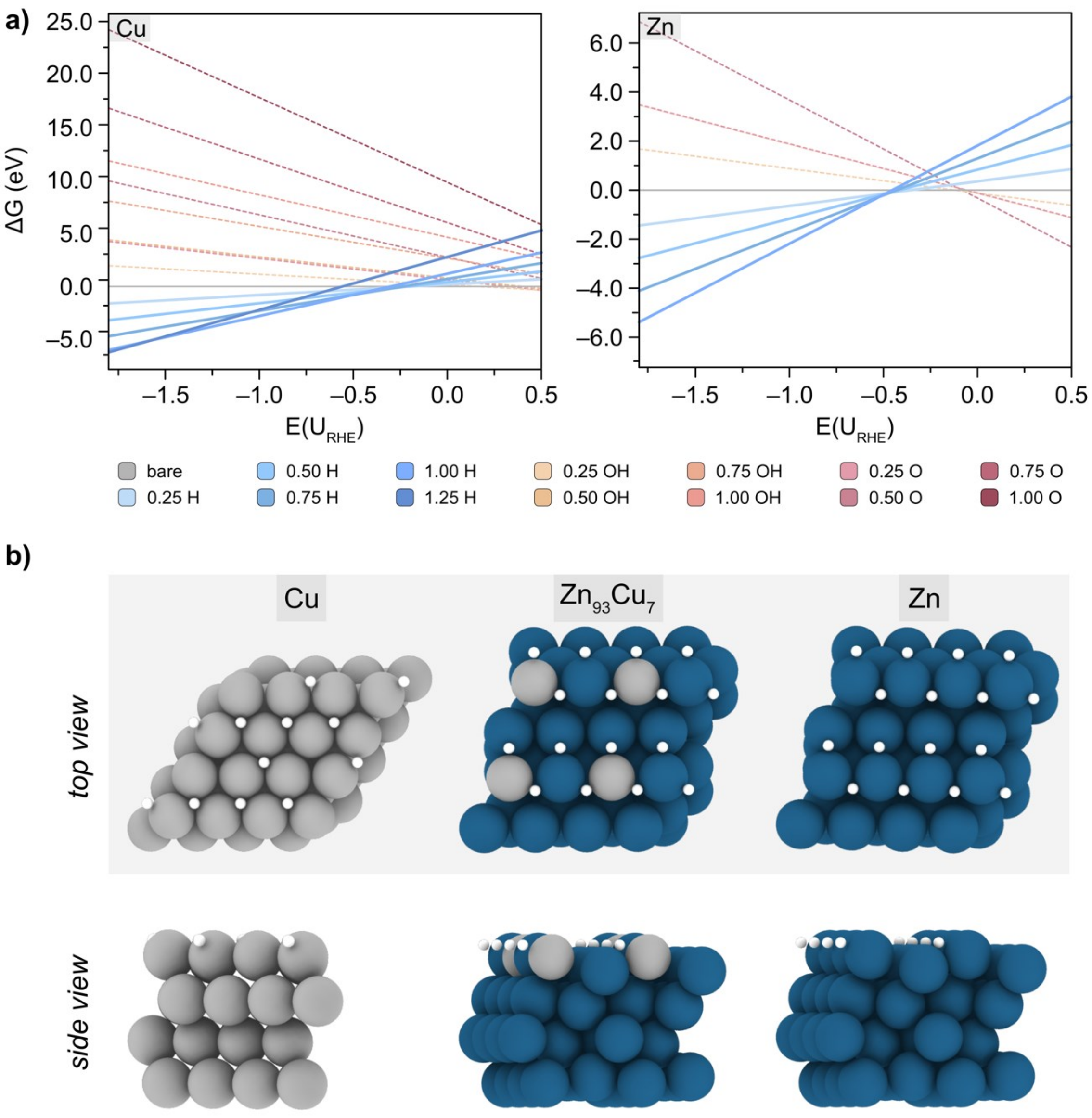

Figure S17. Computational details for the surface coverage of electrocatalysts. a) Plots of ΔG_{*X} of *H, *OH and *O groups on the Cu(111) and Zn(101) *p*(2×2) supercells, as a function of the applied potential expressed versus the reversible hydrogen electrode, $E(V_{RHE})$. The blue lines indicate H coverages, while the dotted red lines indicate O and OH coverages. The coverage densities are expressed as outlined in the **Computation details** section. b) From left to right: top and side views of the resting states of the Cu(111), $Zn_{93}Cu_7$(101) and Zn(101) surface slabs featuring the most stable coverage under reaction conditions, *i.e*. a H coverage of 75% and 100% of the *fcc* sites, respectively. Color code: Cu (grey), Zn (blue), H (white).

References

(1) Wu, Y.; Jiang, Z.; Lin, Z.; Liang, Y.; Wang, H. Direct electrosynthesis of methylamine from carbon dioxide and nitrate. *Nat. Sustain.* **2021**, *4* (8), 725-730. DOI: 10.1038/s41893-021-00705-7.

(2) Fortune, W. B.; Mellon, M. G. Determination of Iron with o-Phenanthroline: A Spectrophotometric Study. *Ind. Eng. Chem. Anal. Ed.* **1938**, *10* (2), 60-64. DOI: 10.1021/ac50118a004.

(3) Kresse, G.; Furthmüller, J. Efficient iterative schemes for ab initio total-energy calculations using a plane-wave basis set. *Phys. Rev. B* **1996**, *54* (16), 11169-11186. DOI: 10.1103/PhysRevB.54.11169.

(4) Wellendorff, J.; Lundgaard, K. T.; Møgelhøj, A.; Petzold, V.; Landis, D. D.; Nørskov, J. K.; Bligaard, T.; Jacobsen, K. W. Density functionals for surface science: Exchange-correlation model development with Bayesian error estimation. *Phys. Rev. B* **2012**, *85* (23), 235149. DOI: 10.1103/PhysRevB.85.235149.

(5) Blöchl, P. E. Projector augmented-wave method. *Phys. Rev. B* **1994**, *50* (24), 17953-17979. DOI: 10.1103/PhysRevB.50.17953.

(6) Methfessel, M.; Paxton, A. T. High-precision sampling for Brillouin-zone integration in metals. *Phys. Rev. B* **1989**, *40* (6), 3616-3621. DOI: 10.1103/PhysRevB.40.3616.

(7) Heyden, A.; Bell, A. T.; Keil, F. J. Efficient methods for finding transition states in chemical reactions: comparison of improved dimer method and partitioned rational function optimization method. *J. Chem. Phys.* **2005**, *123* (22), 224101. DOI: 10.1063/1.2104507 From NLM.

(8) Henkelman, G.; Uberuaga, B. P.; Jónsson, H. A climbing image nudged elastic band method for finding saddle points and minimum energy paths. *J. Chem. Phys.* **2000**, *113* (22), 9901-9904. DOI: 10.1063/1.1329672.

(9) Brogaard, R. Y.; Wang, C.-M.; Studt, F. Methanol–Alkene Reactions in Zeotype Acid Catalysts: Insights from a Descriptor-Based Approach and Microkinetic Modeling. *ACS. Catal.* **2014**, *4* (12), 4504-4509. DOI: 10.1021/cs5014267.

(10) Hjorth Larsen, A.; Jørgen Mortensen, J.; Blomqvist, J.; Castelli, I. E.; Christensen, R.; Dułak, M.; Friis, J.; Groves, M. N.; Hammer, B.; Hargus, C.; Hermes, E. D.; Jennings, P. C.; Bjerre Jensen, P.; Kermode, J.; Kitchin, J. R.; Leonhard Kolsbjerg, E.; Kubal, J.; Kaasbjerg, K.; Lysgaard, S.; Bergmann Maronsson, J.; Maxson, T.; Olsen, T.; Pastewka, L.; Peterson, A.; Rostgaard, C.; Schiøtz, J.; Schütt, O.; Strange, M.; Thygesen, K. S.; Vegge, T.; Vilhelmsen, L.; Walter, M.; Zeng, Z.; Jacobsen, K. W. The atomic simulation environment-a Python library for working with atoms. *J Phys Condens Matter* **2017**, *29* (27), 273002. DOI: 10.1088/1361-648X/aa680e From NLM.

(11) Jain, A.; Ong, S. P.; Hautier, G.; Chen, W.; Richards, W. D.; Dacek, S.; Cholia, S.; Gunter, D.; Skinner, D.; Ceder, G.; Persson, K. A. Commentary: The Materials Project: A materials genome approach to accelerating materials innovation. *APL Materials* **2013**, *1*, 011002. DOI: 10.1063/1.4812323.

(12) Birch, F. Finite Elastic Strain of Cubic Crystals. *Phys. Rev.* **1947**, *71* (11), 809-824. DOI: 10.1103/PhysRev.71.809.

(13) Hong, H. L.; Wang, Q.; Dong, C.; Liaw, P. K. Understanding the Cu-Zn brass alloys using a short-range-order cluster model: significance of specific compositions of industrial alloys. *Sci. Rep.* **2014**, *4* (1), 7065. DOI: 10.1038/srep07065.

(14) Nørskov, J. K.; Rossmeisl, J.; Logadottir, A.; Lindqvist, L.; Kitchin, J. R.; Bligaard, T.; Jónsson, H. Origin of the Overpotential for Oxygen Reduction at a Fuel-Cell Cathode. *J. Phys. Chem. B* **2004**, *108* (46), 17886-17892. DOI: 10.1021/jp047349j.

(15) Ciotti, A.; García-Melchor, M. The importance of surface coverages in the rational design of electrocatalysts. *Curr. Opin. Electrochem.* **2023**, 101402. DOI: 10.1016/j.coelec.2023.101402.

(16) Kristinsdóttir, L.; Skúlason, E. A systematic DFT study of hydrogen diffusion on transition metal surfaces. *Surf. Sci.* **2012**, *606* (17), 1400-1404. DOI: 10.1016/j.susc.2012.04.028.

(17) Li, T.; Ciotti, A.; Rahaman, M.; Yeung, C. W. S.; García-Melchor, M.; Reisner, E. Driving electrochemical organic hydrogenation on metal catalysts by tailoring hydrogen surface coverages. *ChemRxiv* **2023**.

(18) Xu, H.; Ma, Y.; Chen, J.; Zhang, W.-x.; Yang, J. Electrocatalytic reduction of nitrate – a step towards a sustainable nitrogen cycle. *Chem. Soc. Rev.* **2022**, *51* (7), 2710-2758. DOI: 10.1039/D1CS00857A.

(19) Hu, T.; Wang, C.; Wang, M.; Li, C. M.; Guo, C. Theoretical Insights into Superior Nitrate Reduction to Ammonia Performance of Copper Catalysts. *ACS. Catal.* **2021**, *11* (23), 14417-14427. DOI: 10.1021/acscatal.1c03666.

(20) Liu, J.-X.; Richards, D.; Singh, N.; Goldsmith, B. R. Activity and Selectivity Trends in Electrocatalytic Nitrate Reduction on Transition Metals. *ACS. Catal.* **2019**, *9* (8), 7052-7064. DOI: 10.1021/acscatal.9b02179.

(21) Wu, Y.; Chen, W.; Jiang, Y.; Xu, Y.; Zhou, B.; Xu, L.; Xie, C.; Yang, M.; Qiu, M.; Wang, D.; Liu, Q.; Liu, Q.; Wang, S.; Zou, Y. Electrocatalytic synthesis of nylon-6 precursor at almost 100% yield. *Angew. Chem. Int. Ed.* **2023**, e202305491. DOI: 10.1002/anie.202305491.

(22) Chen, F. Y.; Wu, Z. Y.; Gupta, S.; Rivera, D. J.; Lambeets, S. V.; Pecaut, S.; Kim, J. Y. T.; Zhu, P.; Finfrock, Y. Z.; Meira, D. M.; King, G.; Gao, G.; Xu, W.; Cullen, D. A.; Zhou, H.; Han, Y.; Perea, D. E.; Muhich, C. L.; Wang, H. Efficient conversion of low-concentration nitrate sources into ammonia on a Ru-dispersed Cu nanowire electrocatalyst. *Nat Nanotechnol* **2022**, *17* (7), 759-767. DOI: 10.1038/s41565-022-01121-4.
